# Alterations in Excitatory and Inhibitory Synaptic Development Within the Mesolimbic Dopamine Pathway in a Mouse Model of Prenatal Drug Exposure

**DOI:** 10.3389/fped.2021.794544

**Published:** 2021-12-13

**Authors:** Taylor Boggess, James C. Williamson, Ethan B. Niebergall, Hannah Sexton, Anna Mazur, Richard D. Egleton, Lawrence M. Grover, W. Christopher Risher

**Affiliations:** Joan C. Edwards School of Medicine, Marshall University, Huntington, WV, United States

**Keywords:** opioids, synapses, neonatal abstinence syndrome (NAS), gabapentin, alpha-2-delta-1

## Abstract

The rise in rates of opioid abuse in recent years in the United States has led to a dramatic increase in the incidence of neonatal abstinence syndrome (NAS). Despite improved understanding of NAS and its acute symptoms, there remains a paucity of information regarding the long-term effects of prenatal exposure to drugs of abuse on neurological development. The primary goal of this study was to investigate the effects of prenatal drug exposure on synaptic connectivity within brain regions associated with the mesolimbic dopamine pathway, the primary reward pathway associated with drug abuse and addiction, in a mouse model. Our secondary goal was to examine the role of the Ca^+2^ channel subunit α2δ-1, known to be involved in key developmental synaptogenic pathways, in mediating these effects. Pregnant mouse dams were treated orally with either the opioid drug buprenorphine (commonly used in medication-assisted treatment for substance use patients), gabapentin (neuropathic pain drug that binds to α2δ-1 and has been increasingly co-abused with opioids), a combination of both drugs, or vehicle daily from gestational day 6 until postnatal day 11. Confocal fluorescence immunohistochemistry (IHC) imaging of the brains of the resulting wild-type (WT) pups at postnatal day 21 revealed a number of significant alterations in excitatory and inhibitory synaptic populations within the anterior cingulate cortex (ACC), nucleus accumbens (NAC), and medial prefrontal cortex (PFC), particularly in the buprenorphine or combinatorial buprenorphine/gabapentin groups. Furthermore, we observed several drug- and region-specific differences in synaptic connectivity between WT and α2δ-1 haploinsufficient mice, indicating that critical α2δ-1-associated synaptogenic pathways are disrupted with early life drug exposure.

## Introduction

Neonatal abstinence syndrome (NAS) is a collection of signs and symptoms commonly observed in the newborns of mothers who abused certain types of drugs during their pregnancy. The developing fetus acquires a physiological dependence on these drugs and, after being separated from the supply of drug at birth, the infant soon displays the symptoms of withdrawal, which can include irritability, tremors, excessive crying, poor feeding, diarrhea, and, in some of the more severe cases, seizures (1). The rise in the incidence rate of NAS in recent years (2, 3) corresponds with the rise in rates of opioid abuse among pregnant women (4, 5) and the United States in general (6). Considerable research efforts have been made to increase the understanding of the pathophysiology of NAS as well as methods to improve treatment of NAS symptoms (1, 7–10). However, knowledge concerning the long-term effects of prenatal exposure to drugs of abuse on neurological development is limited (11).

Opioids remain one of the most widespread and commonly abused classes of drug among the mothers of NAS patients. One of the most commonly used opioid drugs among pregnant substance abuse patients is buprenorphine, a partial μ-opioid receptor agonist that is often prescribed to replace and prevent the reinforcing effects of more harmful and addictive opioid drugs, such as heroin or fentanyl. Pregnant mothers who are addicted to opioids are commonly enrolled in medication-assisted treatment (MAT), which combines less-addictive prescription opioid drugs—namely methadone or buprenorphine—with behavioral counseling in order to treat addiction and achieve better health outcomes for the mother and child. However, emerging evidence asserts that any opioid use during pregnancy, even when part of a medical treatment plan, can have deleterious effects on the developing fetus (12, 13).

While the increased prevalence of opioid abuse is often credited with the rise in incidence of NAS, it is important to note that many mothers of NAS patients abuse multiple drugs besides, or in combination with, opioids. One prescription drug that has been shown to be increasingly co-abused along with opioids is gabapentin, an anticonvulsant drug also prescribed for the treatment of neuropathic pain (14). Gabapentin was initially believed to have no potential for abuse or addiction (14, 15). However, surveys of opioid use disorder patients have found that as many as 26% of those interviewed reported abusing gabapentin for nonmedical reasons, often abusing gabapentin in combination with opioids as a means to potentiate the experienced high (14, 16, 17). In addition, clinicians have observed a unique presentation of NAS in infants whose mothers abused both opioids and gabapentin while pregnant, with symptoms including tongue thrusting, back arching, and increased eye wandering (18).

**Figure 1:**
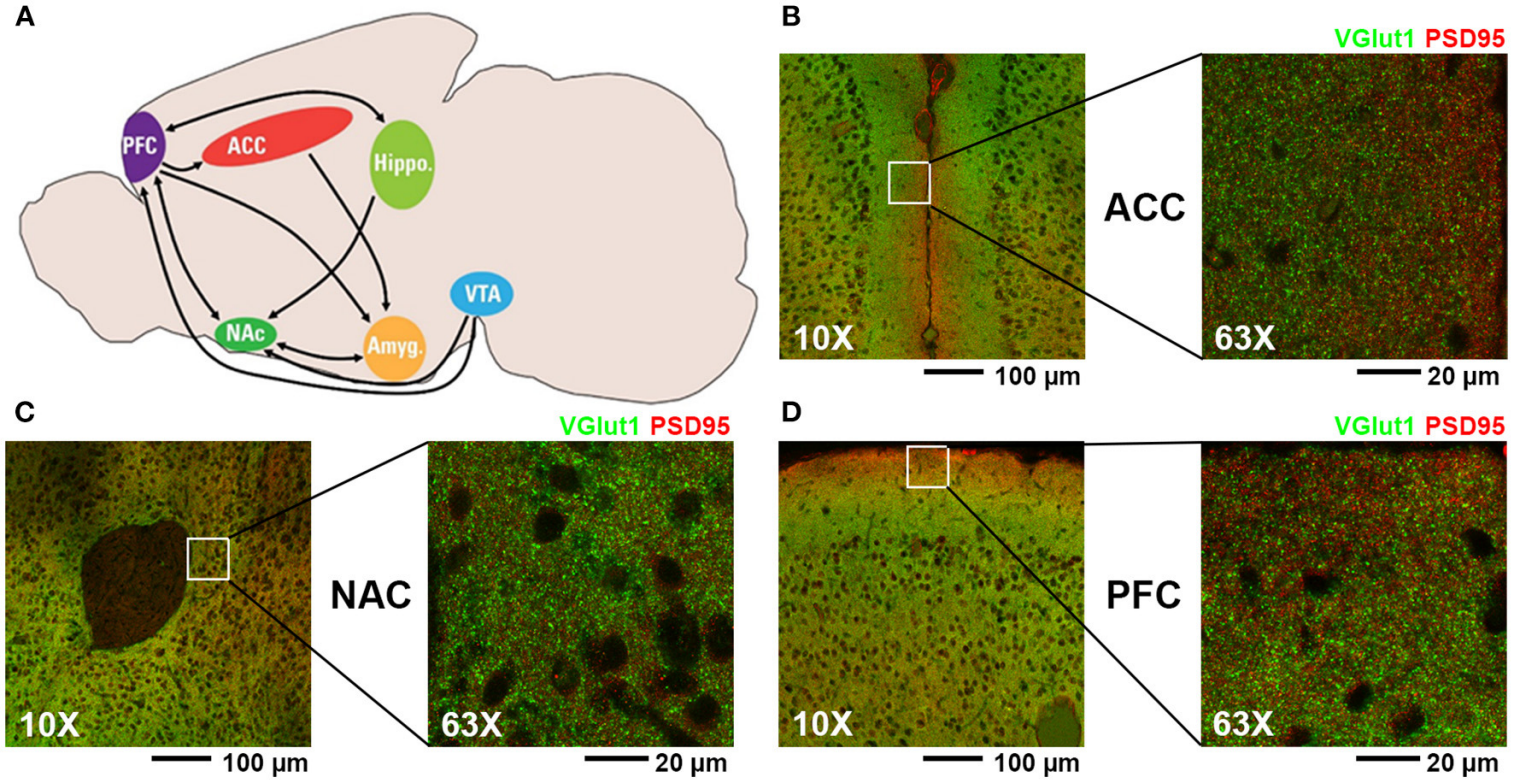
**(A)** Diagram of mouse brain regions associated with mesolimbic dopamine pathway (PFC, prefrontal cortex; ACC, anterior cingulate cortex; NAC, nucleus accumbens; VTA, ventral tegmental area; Hippo, hippocampus; Amyg, amygdala). **(B–D)** Representative fluorescent confocal microscopic images of excitatory synaptic staining (presynaptic VGlut1, green; postsynaptic PSD95, red) at 10X and 63X magnification of the regions analyzed in this study: **(B)** ACC, **(C)** NAC, and **(D)** PFC.

Although the mechanisms by which gabapentin and opioids may interact with each other are unknown, the addictive nature of such drugs of abuse can largely be attributed to their activity within the mesolimbic dopamine pathway (Figure 1) (19–21). Also known as the dopamine reward pathway, this pathway functions by encoding certain experiences (e.g., food, sex, drugs) as pleasurable, then reinforcing future behaviors in an attempt to seek out those same rewarding experiences. Opioids are known to interact with this reward pathway by binding to opioid receptors on the surfaces of interneurons within the ventral tegmental area (VTA), a central location for the cell bodies of a large number of dopaminergic neurons. This opioid-receptor interaction inhibits the release of GABA within the VTA, thus disinhibiting dopamine release by dopaminergic neurons (22). The axons of VTA dopaminergic neurons project to the nucleus accumbens (NAC), where increased phasic release of dopamine in response to external stimuli has been shown to reinforce pleasurable behaviors including eating food, engaging in sexual activity, or taking drugs of abuse (23). The anterior cingulate cortex (ACC) is involved in a variety of functions, including reward anticipation and modulation of goal-oriented motor activity (24, 25) and has been shown to influence motivation via interaction with the mesolimbic dopamine pathway (26), with projections from the VTA having been shown to release dopamine within the ACC in response to microinjections of a μ-opioid receptor agonist (27). The prefrontal cortex (PFC) is highly associated with decision making and impulse control and has been shown to have axonal projections to the NAC capable of modulating neuronal activity within this region (28). Projections from the medial PFC to the NAC have been shown to be involved in reinstatement of drug-seeking behavior in response to stress or drug-associated cues (29, 30).

Examining the effects of drugs of abuse (specifically opioids and gabapentin) on synaptogenesis within brain regions associated with the mesolimbic dopamine pathway may lead to insight on how these drugs affect neurological development in prenatally exposed children. In this study, we used fluorescence immunohistochemistry (IHC) in a mouse model of early life drug exposure to investigate the effects of both buprenorphine and gabapentin on developmental synaptic connectivity. Our hypothesis was that exposure to buprenorphine, gabapentin, or a combination of both drugs during critical periods for synaptic development would lead to significant disruptions in excitatory and inhibitory synaptic connectivity in brain regions associated with addiction and reward. We further hypothesized that haploinsufficiency of the calcium channel subunit α2δ-1, the receptor for gabapentin and a critical player in developmental synapse formation and maturation, would result in further disruption of these synaptic populations.

## Materials and Methods

### Animals and Drug Treatment

All experiments were conducted in accordance with Marshall University's Institutional Animal Care and Use Committee (IACUC) guidelines (W.C.R. protocols 696 and 697). Adult C57/Bl6 mice heterozygous for *Cacna2d1* (i.e. α2δ-1 +/-) (31) were a kind gift from Dr. Cagla Eroglu (Duke University). The mice were bred onsite at the Marshall University Animal Resource Facility. Virgin α2δ-1 +/- females and α2δ-1 +/- males were set up as mating pairs. Upon visual confirmation of the vaginal plug (established as embryonic day 0 [E0]), males and females were separated. On E6, pregnant females were given access to 1 ml of a 1:1 sweetened condensed milk/water solution served in a plastic 35 mm dish. Starting on E7, pregnant females were given free access to a once daily 1 ml solution of 1:1 condensed milk/water containing either pharmaceutical grade buprenorphine hydrochloride (CIII) (5 mg/kg; Spectrum Chemical, Gardena, CA), gabapentin (30 mg/kg; Spectrum), a combination of both drug doses, or vehicle control. Drug doses were calculated based on the weight of pregnant females on E7. Daily dosing continued through the birth of pups and ended on postnatal day 11 (P11). All animals were given *ad libitum* food (standard mouse chow milled on site) and water.

For wild-type (WT) mice, 7 pups (3 male and 4 female) from vehicle control treated litters, 6 (2 male, 4 female) from gabapentin treated litters, 6 (3 male, 3 female) from buprenorphine treated litters, and 7 (4 male, 3 female) from combined buprenorphine and gabapentin treated litters were included. For α2δ-1 +/- mice, 11 pups (5 male, 6 female) from vehicle control treated litters, 7 (3 male, 4 female) from gabapentin treated litters, 7 (4 male, 3 female) from buprenorphine treated litters, and 7 (4 male, 3 female) from combined buprenorphine and gabapentin treated litters were included.

### Mouse Brain Sample Drug Concentration Analysis

The brains of newborn WT pups from dams prenatally treated with 5 mg/kg buprenorphine, 30 mg/kg gabapentin, or vehicle control (2 litters per treatment) were isolated and then shipped on dry ice to the Pharmaceutical Sciences Research Institute at the McWhorter School of Pharmacy (Samford University, Birmingham, AL) for drug concentration analysis. Mouse brain samples were homogenized 1:5 with 5 mM ammonium acetate buffer using an Omni THQ homogenizer (Atlanta, GA). Gabapentin calibration standards, blanks and QCs were prepared by spiking 20 μl of blank control brain homogenate with the appropriate amount of gabapentin to achieve concentrations in ranging from 25–10,000 ng/ml. Internal standard (1 ml of 100 ng/ml Gabapentin-d4 in methanol) was added to precipitate the proteins. After centrifugation for 5 min at 13,000 rpm, the supernatant was transferred to glass tubes and the solvent was evaporated under nitrogen at 40°C. The samples were then redissolved in 200 μl of 95/5 10 mM ammonium formate/methanol with 0.1% formic acid and transferred to limited volume autosampler vials and analyzed in positive ion mode by liquid chromatography with tandem mass spectrometry (LC/MS/MS).

Buprenorphine calibration standards, blanks, and quality controls (QCs) were prepared by spiking 100 μl of blank control brain homogenate with the appropriate amount of buprenorphine to achieve concentrations ranging from 0.2–200 ng/ml. Standards, blanks, QCs and samples were spiked with internal standard (10 μl of 50 ng/ml Terfenadine in acetonitrile). Acetonitrile (1 ml) was added to precipitate the proteins. After centrifugation for 5 min at 13,000 rpm, the supernatant was transferred to glass tubes and the solvent was evaporated under nitrogen at 50°C. The samples were then redissolved in 200 μl of 50/50 5 mM ammonium acetate/acetonitrile and transferred to limited volume autosampler vials and analyzed in positive ion mode by LC/MS/MS.

The LC/MS/MS system consisted of Shimadzu system (Columbia, MD) equipped with LC20-AD dual HLPC pumps, an SIL20-AC HT autosampler, and a DGU-20A2 in-line degasser. Detection was performed using an Applied BioSystems 4000 QTRAP (Applied Biosystems, Foster City, CA) triple quadrupole mass spectrometer operated in the positive ion mode utilizing electrospray ionization. Mass calibration, data acquisition and quantitation were performed using Applied Biosystem Analyst 1.6.2 software (Applied Biosystems).

Separation of gabapentin and the gabapentin-d4 from the brain homogenate matrix was achieved from a 10 μl injection of the samples using a Phenomenex Polar C18, 100 X 2 mm 5 μm particle column. The mobile phase was delivered at a flow rate of 400 μl/min using a gradient elution profile consisting of 5 mM ammonium acetate (A) and acetonitrile with 0.1% formic acid (B). The analyte and internal standard were detected using multiple reaction monitoring (MRM) for the following transitions: Gabapentin (m/z 172.2 → 137.0), Gabapentin-d4 (m/z 176.2 → 141.0).

Separation of buprenorphine and terfenadine from the brain homogenate matrix was achieved from a 10 μl injection of the samples using a Phenomenex Luna C18, 100 X 2 mm 5 μm particle column. The mobile phase was delivered at a flow rate of 400 μl/min using a gradient elution profile consisting of 5 mM ammonium acetate with 0.1% formic acid (A) and acetonitrile with 0.1% formic acid (B). The analyte and internal standard were detected using multiple reaction monitoring (MRM) for the following transitions: Buprenorphine (m/z 468.4 → 396.1), Terfenadine (m/z 472.4 → 436.3). Drug concentrations are summarized in Table 1, with extended summary data available online at Harvard Dataverse (https://doi.org/10.7910/DVN/QKL6OP).

**Table 1:** Drug concentrations (shown as mean ± SEM) within brain tissue lysates from drug-exposed newborn mouse pups determined via liquid chromatography-tandem mass spectrometry.

**Drug concentrations in brain tissue lysates from exposed pups**				
**Treatment**	**Maternal dose**	**n**	**Concentration**	**Pup:Maternal Conc. Ratio**
Buprenorphine	5 mg/kg	6	1.85 ± 0.30 nM	0.054 ± 0.009
Gabapentin	30 mg/kg	15	3.29 ± 0.84 μM	0.259 ± 0.037
Vehicle	0 mg/kg	15	0 μM (both drugs)	N/A

### Genotyping

At P7, the tails of the pups were clipped and collected. Toe pads were tattooed (AIMS NEO9 Animal Tattoo System, Hornell, NY) for later identification. Tissue digestion was performed on the clipped tails and DNA was isolated for PCR amplification and genotyping with a 2% agarose gel. Pups were identified as either α2δ-1 +/- heterozygous, α2δ-1 +/+ wildtype (WT), or α2δ-1 -/- knockout (KO) using the following primers: F1 (WT forward, 5′-TCTCAGTTACAAGACTATGTGG-3′), F3 (KO forward, 5′-GGCTGTGTCCTTATTTATGG-3′), and LAF-Test (reverse, 5′-AGTAGGAGAAGGTACAATCGGC-3′) (Integrated DNA Technologies, Coralville, IA).

### Perfusion, Freezing, and Cryosectioning

At P21, the dam and pups were placed on a scale to be weighed (group data is summarized in Table 2) before being anesthetized with tribromoethanol/Avertin (250 mg/kg). Cardiac perfusion was performed, first with 0.24 μg/ml heparin salt in 0.1M phosphate buffered saline (PBS) for 2 min followed by 4% paraformaldehyde (PFA) for 2–5 min at a flow rate of 5 ml/min. The brains of the dam and pups were extracted and submerged in 4% PFA at 4°C for 24 h. After 24 h, the brains were rinsed in PBS and then placed in 30% sucrose:PBS solution for 48 h. Brains in sucrose solution were frozen within plastic embedding molds (Cat. #70182, Electron Microscopy Sciences, Hatfield, PA) in 2:1 sucrose solution:Tissue Freezing Medium (Cat. #72592-G, Electron Microscopy Sciences) and stored at −80°C. Brains were then cryosectioned using a Leica CM 1950 (Leica, Wetzlar, Germany) to 20 μm slices and stored in 50% glycerol:Tris-Buffered Saline (TBS) at −20°C.

**Table 2:** Body mass of dams and pups (shown as mean ± SEM) in each treatment and genotype group at postnatal day 21 perfusion date.

**Body mass of mice at P21 sacrifice date**									
	**Treatment**	**Vehicle control**		**30 mg/kg gabapentin**		**5 mg/kg buprenorphine**		**5 mg/kg Buprenorphine** **+ 30 mg/kg gabapentin**	
Wildtype (WT)									
	Sex	M (*n =* 3)	F (*n =* 4)	M (*n =* 2)	F (*n =* 4)	M (*n =* 3)	F (*n =* 3)	M (*n =* 4)	F (*n =* 3)
	Mass (g)	10.00 ± 0.12	9.03 ± 0.22	9.00 ± 0.35	9.98 ± 0.38	7.20 ± 0.62	7.80 ± 0.31	11.73 ± 0.31	12.05 ± 0.09
Heterozygous (α2δ-1 +/-)									
	Sex	M (*n =* 5)	F (*n =* 6)	M (*n =* 3)	F (*n =* 4)	M (*n =* 4)	F (*n =* 3)	M (*n =* 4)	F (*n =* 3)
	Mass (g)	10.15 ± 0.25	9.33 ± 0.14	10.3 ± 0.55	9.53 ± 0.25	8.33 ± 0.11	8.60 ± 0.07	12.50 ± 0.06	12.07 ± 0.13
	**Treatment**	**Vehicle control (*****n =*** **4)**		**30 mg/kg gabapentin (*****n =*** **5)**		**5 mg/kg buprenorphine (*****n =*** **4)**		**5 mg/kg buprenorphine** **+ 30 mg/kg gabapentin (*****n =*** **5)**	
Dams								
	Mass (g)	33.30 ± 0.35		30.63 ± 0.25		31.35 ± 0.48		27.30 ± 0.36	

### Synaptic Labeling Immunohistochemistry

Three independent coronal sections per mouse containing the PFC (bregma, 2.96 to 2.58 mm; interaural, 6.76–6.38 mm) and three sections per mouse containing the ACC and NAC core/shell (bregma, 1.42–0.86 mm; interaural, 5.22–4.66 mm) were used for analyses (32). After selection, brain slices were washed in TBS + 0.2% Triton X-10% (Cat. #11332481001, Roche Diagnostics, Mannheim, Germany) w/v (TBST) solution at room temperature. The slices were then placed in a 5% normal goat serum (NGS; Cat. #005-000-121, Jackson Immuno Research Laboratories Inc., West Grove, PA):TBST solution for 1 h to block nonspecific binding sites. After blocking was completed, the slices were then placed in 5% NGS:TBST containing primary antibodies against both pre- and post-synaptic proteins. Guinea pig anti-vesicular glutamate transporter 1 (VGlut1; Cat. #AB5905, EMD Millipore, Burlington, MA) at 1:2,000 dilution was used to identify excitatory glutamatergic presynaptic axonal regions while rabbit anti-post synaptic density protein 95 (PSD95; Cat. #51-6900, Invitrogen, Carlsbad, CA) at 1:300 dilution was used to identify postsynaptic dendritic regions. To label inhibitory GABAergic synapses, guinea pig anti-vesicular GABA transporter (VGAT; Cat. #131004, Synaptic Systems, Göttingen, Germany) at 1:1,000 dilution was used to identify presynaptic axonal regions and rabbit anti-gephyrin (Cat. #147002, Synaptic Systems) at 1:1,000 dilution was used to identify postsynaptic dendritic regions. Slices were incubated overnight on an orbital shaker at 4°C.

After overnight incubation was completed, brain slices were removed from primary antibody solution and washed with TBST. Slices were then placed in 5% NGS:TBST containing fluorescent secondary antibodies against the primary antibodies. Alexa Fluor IgG (H+L) 594 goat anti-guinea pig (Cat. #A11076, Invitrogen, Carlsbad, CA) at 1:200 dilution was used to stain against presynaptic antibodies (either VGlut1 for excitatory or VGAT for inhibitory) and Alexa Fluor IgG (H+L) 488 goat anti-rabbit (Cat. #A11034, Invitrogen) at 1:200 dilution was used to stain against postsynaptic antibodies (either PSD95 for excitatory or gephyrin for inhibitory). Slices were incubated for 2 h at room temperature in darkness. After incubation was complete, the slices were washed with TBST.

After the final washing step, the slices were rinsed once in 2/3 TBS:1/3 dH_2_0 solution. Then, the slices were transferred onto glass slides and excess liquid was suctioned off with a pipette. 1 drop of mounting media containing DAPI (Cat. #H-1200; VectaShield, Burlingame, CA) was applied to each slice prior to placement of coverslip and sealing with clear nail polish. The glass slides were allowed to dry overnight in darkness before being stored at −20°C.

### Confocal Fluorescence Microscopy for Synaptic Protein Imaging

Images of the slides were captured using the Leica SP5 Confocal Fluorescence Microscope housed and maintained by the Marshall University Molecular Biological and Imaging Core. Argon visible light laser (at 30% final filter power) was used with emission windows set at 493–550 nm for blue excitation and 595–647 nm for green excitation. Leica LAS AF software was used to capture 5 μm z-stacks with 15 steps (0.33 μm distance between each step) within the brain regions of interest (ROI) across all treatment groups (imaged area/scan = 19,036 μm^2^; 63× oil objective, 1.4 NA).

### Synaptic Puncta Image Analysis

The saved images were analyzed using ImageJ (NIH) software. The custom plug-in “ProjectZ_Triple” (available by request) was used to average and convert the 15 separate images of each z-stack into 5 separate maximum projections, each representing a 1 μm “mini-stack”. Puncta Analyzer (Dr. Cagla Eroglu, Duke University, Durham, NC) was then used to quantify the number of discrete puncta for the 493–550 nm wavelength channel (corresponding to PSD95 in brain slices stained for excitatory synapses or Gephyrin for inhibitory synapses), the 595–647 nm wavelength channel (corresponding to VGlut1 in brain slices stained for excitatory synapses or VGAT for inhibitory synapses), and the co-localized puncta, where puncta from both channels overlapped within 4 pixels of each other. These regions of co-localization of pre- and post-synaptic markers were designated as synapses (33). The counted synapses were compiled into spreadsheets (Microsoft Excel, Redmond, WA) and analyzed with GraphPad Prism (San Diego, CA).

### Statistical Analysis

For body mass comparisons (Supplementary Table 1), dams belonging to different treatment groups were compared via One-Way ANOVA with Tukey's multiple comparisons *post hoc* test. For pups, a Three-Way ANOVA (treatment x genotype x sex) was performed with Tukey's multiple comparisons *post hoc* test. The D'Agostino and Pearson omnibus normality test (Supplementary Table 2) confirmed that most synapse counts were not normally distributed; therefore, statistical differences were analyzed using the nonparametric Kruskal-Wallis test to detect differences in mean number of synapses amongst drug treatments or between genotypes. *Post-hoc* Dunn's multiple comparisons tests were performed to detect significant differences (*p* < 0.05) amongst the mean values of each treatment group (Supplementary Tables 3, 4). Analyses were performed using GraphPad Prism.

## Results

### Confirming Drug Accumulation in Neonatal Mouse Brain Tissue

Each day during the dosing period, pregnant mice were administered drug treatments as 1 ml of a 1:1 sweetened condensed milk/water solution served in a plastic dish. Dishes from the previous day were always observed to be empty, confirming that the dams routinely consumed the entirety of the treatment solution. To confirm drug passage from the dam to the pups, the brains of newborn pups from litters prenatally treated with buprenorphine or gabapentin were isolated, lysed, and subjected to liquid chromatography-tandem mass spectrometry to determine the brain tissue concentrations of each drug. Both buprenorphine and gabapentin were detected in the brains of the prenatally exposed pups (Table 1), prompting further investigation of brain connectivity changes as a result of the presence of these compounds.

### Body Mass of Dams and Pups

In order to produce the required number of WT and α2δ-1 heterozygous (+/–) pups for IHC in this study, the following litter numbers were required: 4 litters dosed with vehicle control, 5 litters dosed with 30mg/kg gabapentin, 4 litters dosed with 5mg/kg buprenorphine, and 5 litters dosed with both drugs. Immediately before anesthetization and perfusion at P21, dams and all pups from each litter were weighed and their weights were recorded. The mean masses for the dams of the different litters and the pups in each sex-genotype-treatment group were calculated (Table 2). The mean body masses for dams revealed a significant main effect of treatment, *F*(3, 14) = 50.21, *p* < 0.0001. Dams treated with buprenorphine (*p* = 0.0122), gabapentin (*p* = 0.0006), and buprenorphine + gabapentin (*p* < 0.0001) were all significantly lower than the mean body mass of the vehicle control dams. Significant differences were also observed among the various pup groupings across treatment [*F*(3, 42) = 140.4, *p* < 0.0001], genotype [*F*(1, 42) = 12.66, *p* =0.0009], treatment × sex [*F*(3, 42) = 4.487, *p* = 0.0081], and genotype × sex [*F*(1, 42) = 5.627, *p* = 0.0223]. No significance was found for the main effect of sex [*F*(1, 42) = 0.5268, *p* = 0.4720] or the interactions of treatment × genotype [*F*(3, 42) = 1.301, *p* = 0.2866] or treatment × genotype × sex [*F*(3, 42) = 2.039, *p* = 0.1229]. Full multiple comparisons are listed in Supplementary Table 1. Of note, key differences between treatments that reached significance included: WT male vehicle vs. either WT male buprenorphine (Bup) or WT male buprenorphine plus gabapentin (BupGBP), WT female vehicle vs. WT female BupGBP; heterozygous (Het) male vehicle vs. either Het male Bup or Het male BupGBP, and Het female vehicle vs. Het female BupGBP.

### Early Life Exposure to Buprenorphine Increases Excitatory Synapses in the Mesolimbic Dopamine Pathway

**Figure 2:**
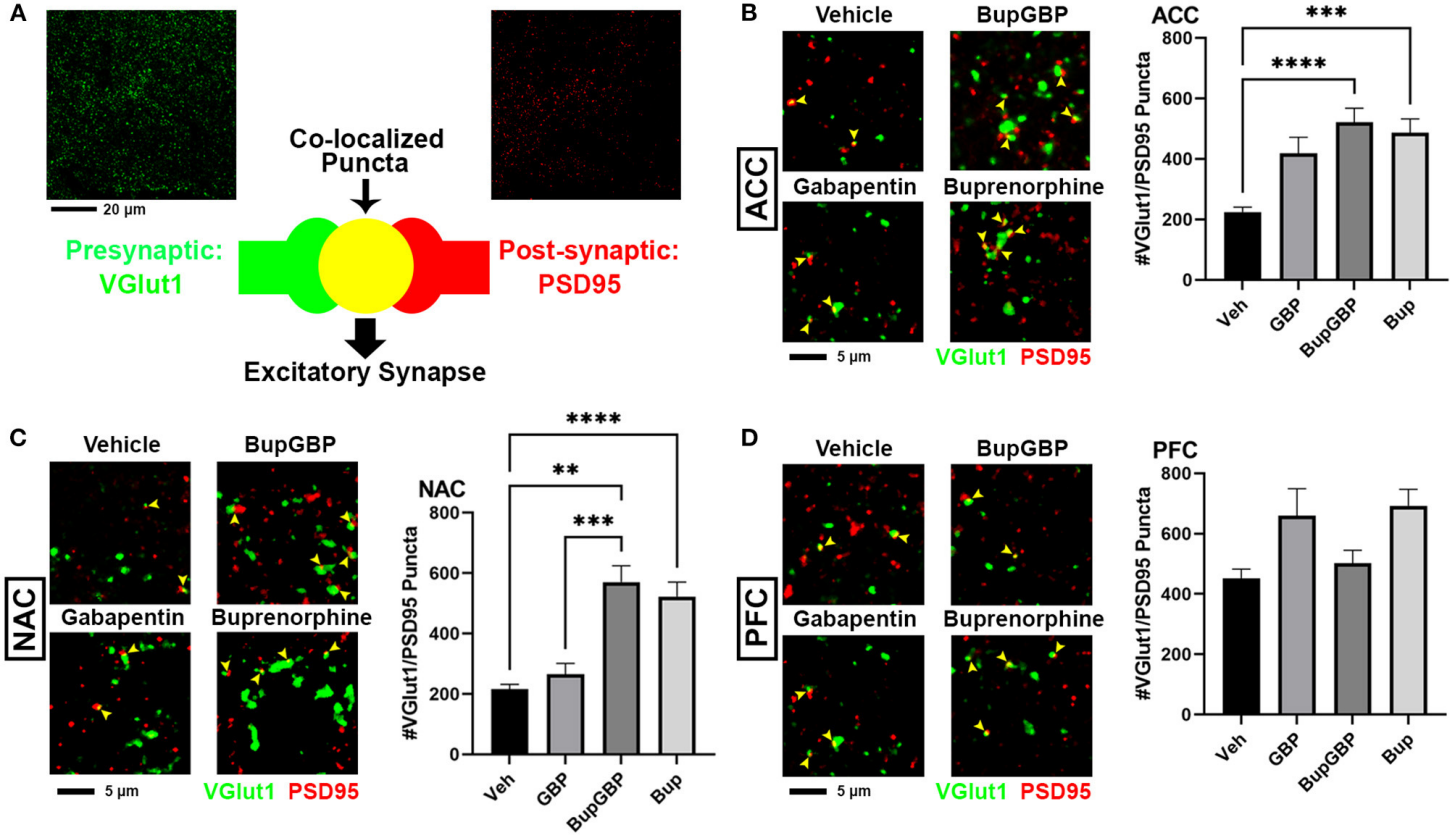
Increased excitatory synapses with early life buprenorphine exposure. **(A)** Diagram illustrating co-localization of puncta representing presynaptic (VGlut1, green) and postsynaptic (PSD95, red) fluorescent antibody label pairs for quantifying excitatory glutamatergic synapses. **(B–D)** Representative IHC images (left) and quantification (right) of co-localized VGlut1 (green) and PSD95 (red) excitatory synaptic puncta (yellow arrowheads) from prenatal drug-exposed WT C57Bl/6J P21 mouse brain within **(B)** the anterior cingulate cortex (ACC), **(C)** nucleus accumbens (NAC), and **(D)** prefrontal cortex (PFC). *n* = 7 (vehicle control), 6 (GBP), 7 (Bup+GBP), 6 (Bup); **p < 0.01; ***p < 0.001; ****p < 0.0001.

We next wanted to determine whether excitatory synaptic development within the mesolimbic dopamine pathway was significantly impacted by the presence of buprenorphine and/or gabapentin *in utero*. Fluorescence IHC followed by confocal imaging was used to distinguish and quantify excitatory glutamatergic synapses within three different brain regions of WT mouse pups at P21 (Figure 2). Synapses were identified by the co-localization of presynaptic VGlut1 and postsynaptic PSD95 (Figure 2A). This type of imaging-based synapse analysis relies on the fact that these markers are expressed in completely different neuronal compartments (i.e., VGlut1 in axons, PSD95 in dendrites), and would only appear co-localized in confocal microscopy when directly opposed at sites of synaptic contact. Indeed, rotating the channels out of alignment with each other results in a dramatic decrease in co-localized puncta (Supplementary Figure 1). Beginning with excitatory glutamatergic synapses within the ACC (Figure 2B), we observed a significant main effect between groups, *H* = 99.00, *p* < 0.0001 (full list of multiple comparisons reported in Supplementary Table 3), as mice treated with either buprenorphine or buprenorphine in combination with gabapentin had a significantly increased mean number of synapses than mice treated with vehicle control. Interestingly, gabapentin treatment on its own did not significantly impact excitatory synapse number compared to vehicle, despite its known role as an inhibitor of prominent synaptogenic pathways (34). Similar results were also observed within the NAC (Figure 2C; *H* = 119.2, *p* < 0.0001), with buprenorphine and buprenorphine together with gabapentin having significantly more synapses than vehicle, with no significant effect of gabapentin alone. However, in the NAC, the combination drug treatment group also exhibited a significantly higher number of synapses than the gabapentin-only group. In contrast, within the PFC (Figure 2D; *H* = 165.0, *p* < 0.0001), no significant differences were observed among any of the treatment groups when compared to vehicle.

### Combined Buprenorphine/Gabapentin Exposure Decreases Inhibitory Synapse Number

**Figure 3:**
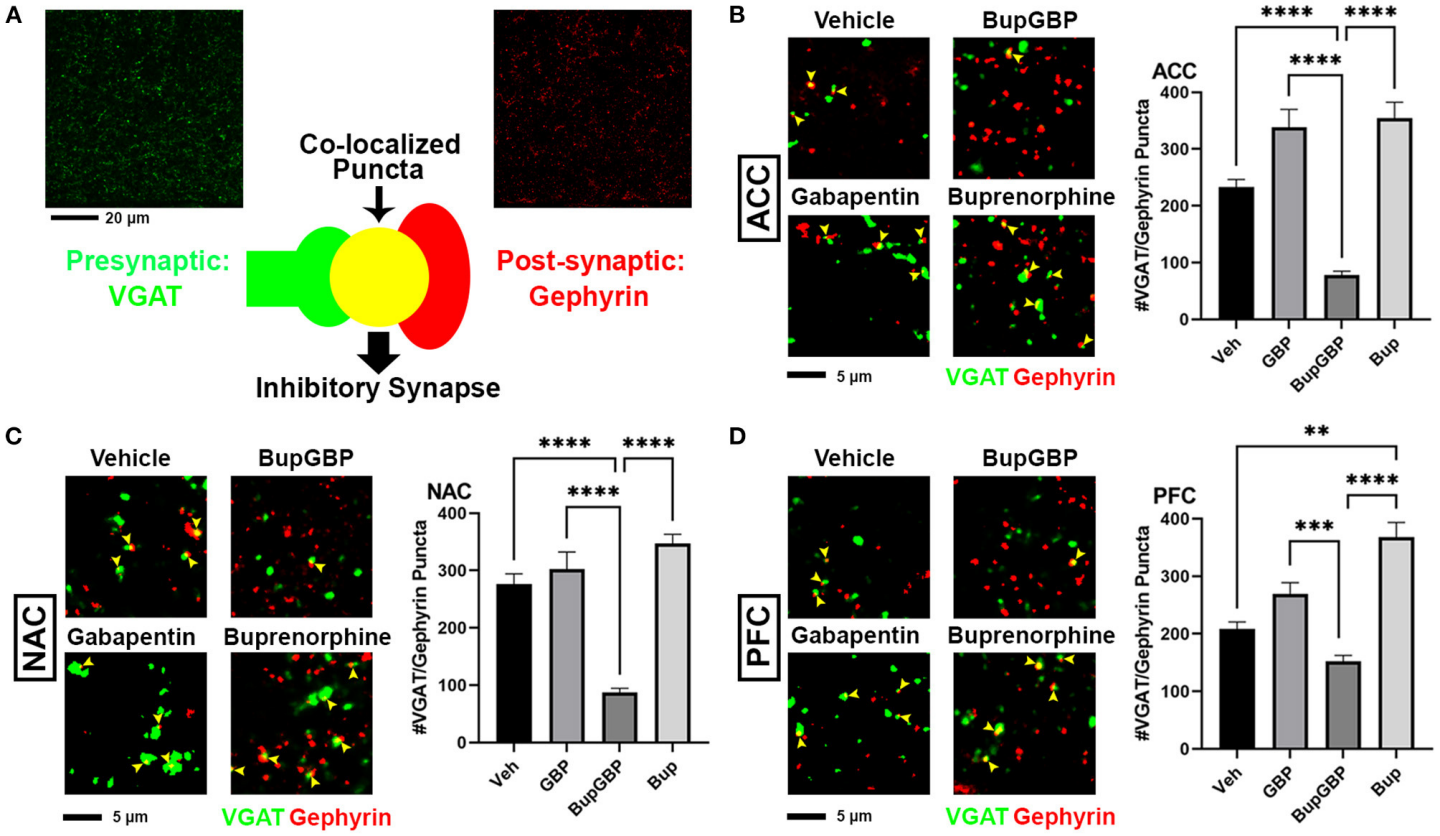
Dual exposure to buprenorphine and gabapentin decreased inhibitory synapses at P21. **(A)** Diagram illustrating co-localization of puncta representing presynaptic (VGAT, green) and postsynaptic (PSD95) fluorescent antibody label pairs for quantifying inhibitory GABAergic synapses. **(B–D)** Representative IHC images (left) and quantification (right) of co-localized VGAT (green) and gephyrin (red) inhibitory synaptic puncta (yellow arrowheads) from prenatal drug-exposed WT C57Bl/6J P21 mouse brain within **(B)** ACC, **(C)** NAC, and **(D)** PFC. *n* = 7 (vehicle control), 6 (GBP), 7 (Bup+GBP), 6 (Bup); **p < 0.01; ***p < 0.001; ****p < 0.0001.

The same IHC procedure used for glutamatergic excitatory synapses was next used to stain for GABAergic inhibitory synapses (identified by the co-localization of presynaptic VGAT and postsynaptic gephyrin; Figure 3A) within the same brain regions of the previously analyzed WT pups. As with excitatory synapses, we observed a main effect of treatment, *H* = 196.5, *p* < 0.0001 (full list of multiple comparisons reported in Supplementary Table 4), for inhibitory synapses within the ACC (Figure 3B). A significant decrease in mean number of inhibitory synapses was observed in the combination drug treated mice compared to all other treatment groups. As with excitatory synapses, similar observations were made in the NAC (Figure 3C; *H* = 219.8, *p* < 0.0001), with the buprenorphine plus gabapentin group having significantly fewer inhibitory synapses than the other groups. The PFC, however, was the only region that exhibited an increase in inhibitory synapse number with any of the drug treatments (Figure 3D; *H* = 121.8, *p* < 0.0001), with the buprenorphine treatment group having significantly more VGAT/gephyrin co-localized puncta than the vehicle control group. Yet, as in the ACC and NAC, the combination treated mice had significantly fewer synapses within the PFC than mice from either of the single drug treatment groups, but not compared to vehicle control.

### α2δ-1 Haploinsufficiency Results in Region- and Treatment-Specific Differences in Synaptic Connectivity

**Figure 4:**
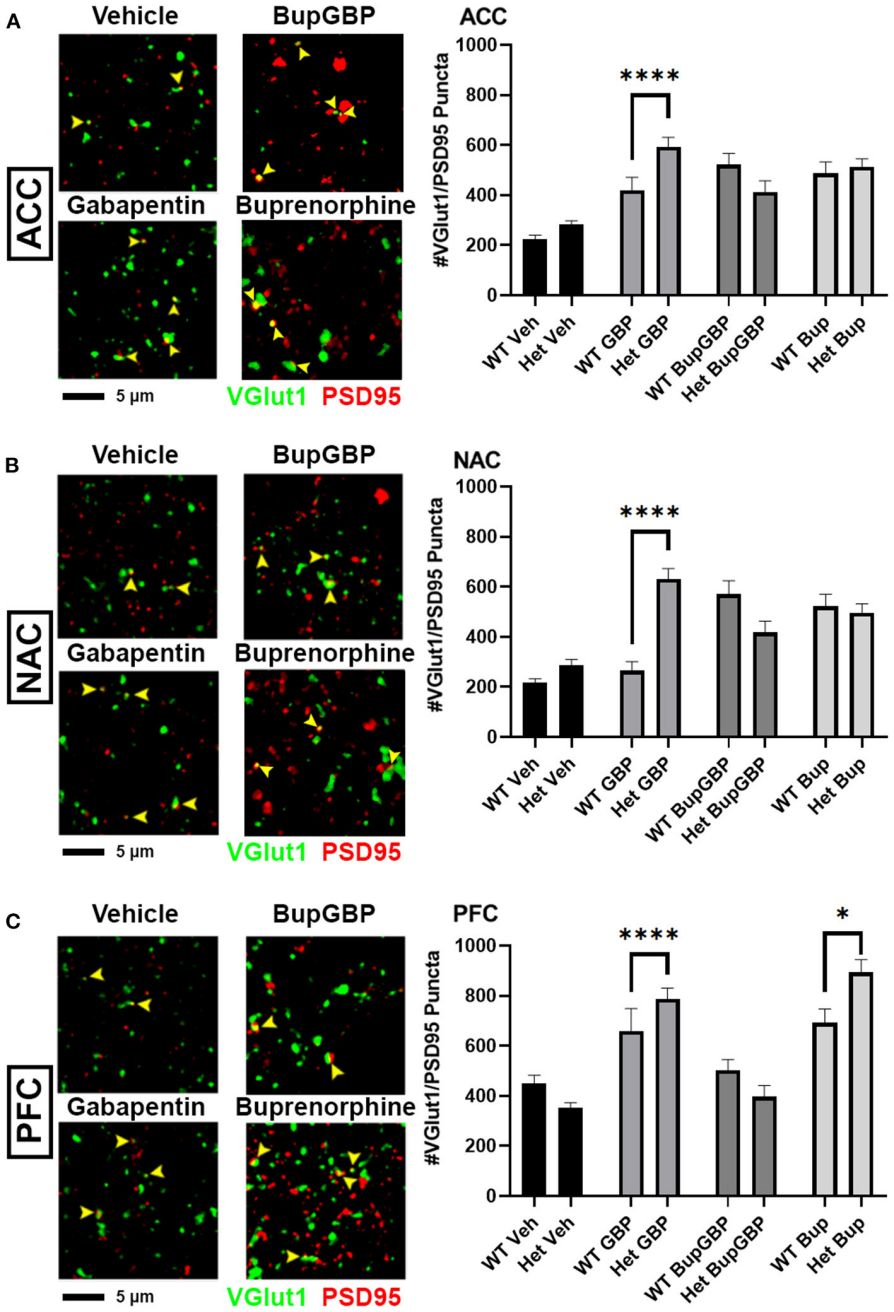
Altered excitatory synaptic connectivity following prenatal drug treatment in α2δ-1 haploinsufficient mice. Representative IHC images (left) of co-localized VGlut1 (green) and PSD95 (red) excitatory synaptic puncta (yellow arrowheads) from prenatal drug-exposed α2δ-1 +/- (Het) C57Bl/6J P21 mouse brain with quantification (right) compared to WT C57Bl/6J P21 mouse brains with the same prenatal treatment within **(A)** ACC, **(B)** NAC, and **(C)** PFC. Het: *n* = 11 (vehicle control), 7 (GBP), 7 (Bup+GBP), 7 (Bup); *p < 0.05; ****p < 0.0001.

Gabapentin has previously been shown to inhibit excitatory synapse formation by binding to the neuronal calcium channel subunit α2δ-1 (34), and this mechanism is proposed to underlie the efficacy of gabapentin in the alleviation of symptoms in both epilepsy and neuropathic pain (35, 36). We previously showed deficits in synaptic connectivity in the brains of young mice lacking α2δ-1 (31). To determine whether α2δ-1-mediated synaptic development is impacted by early life drug exposure, we expanded our synapse analysis to compare our WT synapse numbers with those from mice that were haploinsufficient for α2δ-1. Comparing excitatory synaptic development, within the ACC (Figure 4A; *H* = 99.00, *p* < 0.0001), only the gabapentin treated α2δ-1 +/- mice showed a significantly greater number of synapses compared with WT mice that received the same treatment. A similar gabapentin-induced increase was observed within the NAC (Figure 4B; *H* = 119.2, *p* < 0.0001). The same held true for the α2δ-1 +/- PFC (Figure 4C; *H* = 165.0, *p* < 0.0001), which also exhibited more synapses than WT mice with buprenorphine treatment.

**Figure 5:**
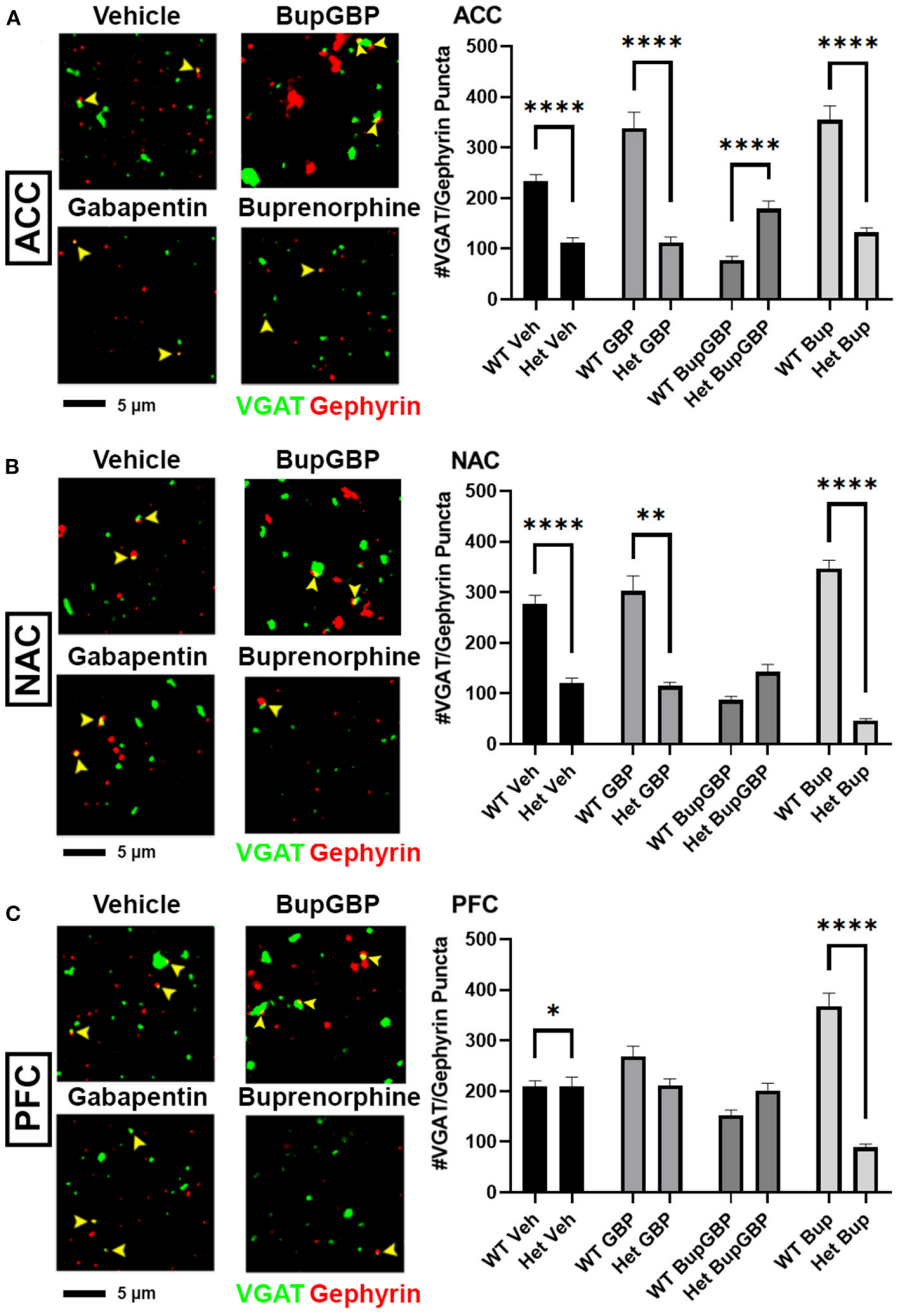
α2δ-1-influenced changes in inhibitory synapse development in drug-exposed pups. Representative IHC images (left) of co-localized VGAT (green) and gephyrin (red) inhibitory synaptic puncta (yellow arrowheads) from prenatal drug-exposed α2δ-1 Het C57Bl/6J P21 mouse brain and quantification (right) compared to WT C57Bl/6J P21 mouse brains with the same prenatal treatment within **(A)** ACC, **(B)** NAC, and **(C)** PFC. Het: *n* = 11 (vehicle control), 7 (GBP), 7 (Bup+GBP), 7 (Bup); *p < 0.05; **p < 0.01; ****p < 0.0001.

Compared to excitatory synaptic development, much less is known about the roles of α2δ-1 and gabapentin in the formation of inhibitory circuits. We concluded our analyses by comparing mean inhibitory synapse numbers in α2δ-1 +/- mice to WT mice across the 4 different treatment groups (Figure 5). In the vehicle control, gabapentin, and buprenorphine groups, α2δ-1 +/- mice had significantly fewer synapses within the ACC compared to their WT counterparts (Figure 5A; *H* = 196.5, *p* < 0.0001). Only in the combination treatment group did we observe a significantly higher number of synapses in the ACC of α2δ-1 +/- mice compared to WT. Within the NAC (Figure 5B; *H* = 219.8, *p* < 0.0001), a similar trend was observed among the vehicle control, gabapentin, and buprenorphine groups as was seen in the ACC, with significantly fewer VGAT/gephyrin co-localized synapses in α2δ-1 +/- mice than WT. However, in the NAC, the α2δ-1 +/- mice in the combination treatment group did not differ significantly from the WT mice. Finally, within the PFC (Figure 5C; *H* = 121.8, *p* < 0.0001), the most dramatic significant difference was observed in the buprenorphine treatment group, with α2δ-1 +/- mice showing significantly fewer inhibitory synapses than WT mice. Taken together, these results indicate that α2δ-1 plays an important regulatory role in both excitatory and inhibitory synaptic development in the context of early life drug exposure.

## Discussion

Glutamatergic signaling has been shown to be critical to many different aspects of opioid abuse and addiction (37). Previous studies have shown that morphine-induced activation of dopaminergic neurons cannot occur within the VTA without glutamatergic modulation (38), and glutamate release within the NAC has been shown to be associated with heroin addiction and reinstatement of heroin-seeking behavior in rats (39). In addition, GABAergic synaptic activity has also been heavily implicated in the acute response to drugs and the reinforcement of drug seeking behavior (40, 41). However, relatively little is known about how exposure to drugs of abuse during critical developmental periods affects the trajectories of these synaptic populations. The goal of this study was to investigate the effects of early life drug exposure on synaptic development within brain regions associated with addiction and reward, as well as to explore the role of the gabapentin receptor, α2δ-1, in these alterations of synaptogenesis. Our results indicate that excitatory and inhibitory synaptic populations are significantly disrupted by prenatal exposure to either the opioid buprenorphine or gabapentin, separately or in combination, and that these disruptions are strongly influenced by α2δ-1 function.

Significant increases in excitatory glutamatergic synapses were observed in multiple regions associated with the mesolimbic dopamine reward pathway in WT mice that had undergone prenatal exposure to only buprenorphine or buprenorphine in combination with gabapentin. Concomitantly, mice exposed to the combination buprenorphine-gabapentin treatment *in utero* showed significant decreases in inhibitory GABAergic synapses compared to the vehicle control group within these same regions. In addition, the combined impact of buprenorphine and gabapentin resulted in significantly lower numbers of inhibitory synapses compared to either single drug treatment in all three brain regions, suggesting a synergistic effect of polysubstance use on synaptic development. Given the increased prevalence of polysubstance abuse in mothers of infants born with NAS, as well as the worsened clinical conditions observed in many of these children (42, 43), this finding is of particular concern. Taken together, these results indicate a net increase in excitatory glutamatergic signaling capability within the mesolimbic dopamine pathway of mice prenatally exposed to buprenorphine and gabapentin. Glutamatergic neurotransmission is heavily implicated in many aspects of drug use, including acute effects, consolidation, craving/seeking, withdrawal, and relapse [reviewed in Heinsbroek et al. (37)]. Whether the synaptic population shifts observed in this study result in greater dopamine release in response to rewarding stimuli, such as drugs of abuse, or a greater risk for developing substance abuse disorder later in life remains to be elucidated.

### Drug Dosing Paradigm Correlates

The dosing for buprenorphine in this study was in line with a study by Martin et al. that showed 5 mg/kg to be capable of producing significant increases in pain threshold in rats (44). The gabapentin dosage was chosen based on the finding that 30 mg/kg gabapentin was sufficient to produce analgesia in mice (45). The dosing schedule, starting on E7 and ending on P11, was chosen to correspond to a period of significant synaptogenesis within mice (46–48), a period which also roughly correlates to the second trimester of human fetal development (49, 50). At birth, a mouse pup is considered to be at a developmental stage similar to that of a human fetus in the late second trimester. Despite the fact that newborn mouse pups are no longer receiving direct exposure to drugs of abuse via the placental blood supply, there were still precedents in the literature for postnatal drug transference to the brains of pups being nursed by drug-exposed dams. Kongstorp et al. demonstrated that buprenorphine accumulates and remains in the brain tissue of newborn rodents for several days following birth (51). In addition, both buprenorphine and gabapentin have been shown to pass from mother to infant via breast milk, albeit in low concentrations compared to the mothers' blood plasma levels (52–54). We confirmed the presence of both buprenorphine and gabapentin in the brains of prenatal drug exposed pups, though the levels in our study were admittedly at the lower ends of the ranges reported in the referenced works. Regardless, the results of this study show that the chosen treatment paradigm was capable of disrupting normal CNS synaptic development in mouse pups. The fact that we detected relatively lower concentrations in our animals may even suggest that the observed deficits may have been further exacerbated if the accumulated drug levels had been higher.

### Gabapentin, α2δ-1, and Astrocytes

Unexpectedly, single drug treatment with gabapentin did not result in any significant differences in excitatory or inhibitory synapses within any of the examined brain regions in the WT. This finding would appear to contradict the findings of previous studies showing that gabapentin inhibits normal excitatory synapse development by interfering with pathways mediated by α2δ-1. Though originally designed as a structural analog for the neurotransmitter GABA, gabapentin does not bind to GABA _A_ or GABA _B_ receptors. It is instead proposed that gabapentin decreases neurotransmitter release from the presynaptic terminal by inhibiting Ca^+2^ influx through L-type channels (55, 56). Gabapentin performs this action by binding to α2δ-1 on the surface of neurons at the site of synaptic terminals (35, 57). α2δ-1 is also the binding site for a class of astrocyte-secreted extracellular matrix glycoproteins known as thrombospondins (TSPs), which promote synapse formation and maturation during early development (31, 34). It may be possible that, despite interference by gabapentin in normal TSP/α2δ-1 mediated synaptic development, the net level of synaptogenesis is maintained via other signaling mechanisms as a means to compensate for the gabapentin-induced decrease in glutamate release.

In an attempt to elucidate the impact of α2δ-1 on synaptic development in the drug exposed brain, the original goal of this study was to mate α2δ-1 +/- males and females to produce pups of all three possible α2δ-1 genotypes, i.e. α2δ-1 +/- (Het), α2δ-1 +/+ (WT), and α2δ-1 -/- (knockout; KO). However, after several attempts to breed α2δ-1 KO pups, we found that such pups were either not being born or did not survive to age P21, particularly in the buprenorphine or gabapentin treatment groups. Given the widespread presence of α2δ-1 throughout the mouse CNS (58, 59), skeletal muscle (60, 61), and cardiac muscle (62), the global knockout of this Ca^2+^ channel subunit, in combination with exposure to drugs of abuse during fetal development, may have been potentially lethal.

Despite the difficulty in generating the necessary number of drug exposed α2δ-1 KO pups for this study, we still observed significant drug treatment effects on synaptic development with the loss of just a single copy of α2δ-1, which may indicate that astrocyte-mediated synaptogenic pathways were disrupted with the drug treatment paradigms. Indeed, it has been previously shown that opioids can directly dysregulate the expression and secretion of synaptogenic TSP by astrocytes (63, 64). Far from being a passive bystander in the CNS, astrocytes have been increasingly shown to influence and maintain the excitatory/inhibitory balance necessary for homeostasis (65). Not only are astrocytes able to mediate both excitatory (47) and inhibitory (66) synaptogenesis, they are also able to mediate synapse elimination via phagocytosis mediated by astrocytic cell surface receptors such as MEGF10 and MERTK (67). Outside of synapse regulation, astrocytes are also capable of both uptake (68–70) and release (71, 72) of glutamate and GABA. μ-opioid receptors have been confirmed on the surface of astrocytes within the hippocampus, NAC, and VTA (73), while morphine and other μ-opioid receptor agonists have been shown to alter DNA synthesis (74) and increase expression of glial fibrillary acidic protein (GFAP), an indicator of activation and reactivity, in astrocytes (75). Greater understanding of the role of astrocytes in maintaining excitatory/inhibitory synaptic balance, coupled with increased awareness of the effects of drugs of abuse on astrocytes during development, may facilitate novel insight into how neurological function may be impacted in cases of early life drug exposure.

## Limitations and Conclusions

In this study, we used a mouse model of early life drug exposure to examine the effects such exposure may have on synaptic development within brain regions traditionally associated with reward and addiction. Though there were key regional differences, an overall increase in glutamatergic excitatory synapses as well as a general decrease in GABAergic inhibitory synapses was observed in the mesolimbic dopamine pathway of mice prenatally exposed to buprenorphine or buprenorphine in combination with gabapentin. Such changes in synapse number may indicate a general increase in net excitation in response to rewarding stimuli. α2δ-1, which binds to gabapentin and is involved in critical synaptogenic pathways mediated by astrocytes, was also shown to have an important role in the changes in synapse formation induced by prenatal drug exposure, as evidenced by the significant differences observed between WT and α2δ-1 haploinsufficient animals. We were not able to determine the effects of prenatal drug exposure on mice completely lacking expression of α2δ-1 (possibly due to increased lethality when combined with drug exposure), nor did our study investigate whether these structural synaptic findings persist to later ages and/or correlate with altered physiological function or behavior. However, this work may serve to inform future studies examining these functional effects as well as more longitudinal studies that may link history of prenatal drug exposure with altered reward circuitry and addiction-like behavior in adolescents and adults.

## Data Availability Statement

The raw data supporting the conclusions of this article will be made available by the authors, without undue reservation.

## Ethics Statement

The animal study was reviewed and approved by Marshall University Institutional Animal Care and Use Committee.

## Author Contributions

TB, RE, LG, and WR designed the study. TB, EN, HS, and AM managed the mouse colony and performed genotyping. TB, JW, and EN conducted the experiments. TB and WR analyzed the data and wrote the manuscript. All authors contributed to the article and approved the submitted version.

## Funding

This research was funded by the John and Polly Sparks Foundation, the Brain and Behavior Research Foundation NARSAD Young Investigator Award 27662, and NIH/NIMH 1 R15 MH126345-01 to WR. Support was also provided by the Marshall University Genomics and Bioinformatics Core, the West Virginia IDeA Network of Biomedical Research Excellence (WV-INBRE) grant (P20GM103434), the COBRE ACCORD grant (1P20GM121299), and the West Virginia Clinical and Translational Science Institute (WV-CTSI) grant (2U54GM104942). The funder was not involved in the study design, collection, analysis, interpretation of data, the writing of this article or the decision to submit it for publication.

## Conflict of Interest

The authors declare that the research was conducted in the absence of any commercial or financial relationships that could be construed as a potential conflict of interest.

## Publisher's Note

All claims expressed in this article are solely those of the authors and do not necessarily represent those of their affiliated organizations, or those of the publisher, the editors and the reviewers. Any product that may be evaluated in this article, or claim that may be made by its manufacturer, is not guaranteed or endorsed by the publisher.

## References

[1] Kocherlakota P. Neonatal abstinence syndrome. Pediatrics. (2014) 134:e547–61. 10.1542/peds.2013-3524 25070299

[2] Patrick SW Schumacher RE Benneyworth BD Krans EE Mcallister JM Davis MM. Neonatal abstinence syndrome and associated health care expenditures: United States, 2000-2009trends in neonatal abstinence syndrome. JAMA. (2012) 307:1934–40. 10.1001/jama.2012.3951 22546608

[3] Patrick SW Davis MM Lehmann CU Cooper WO. Increasing incidence and geographic distribution of neonatal abstinence syndrome: United States 2009 to 2012. J Perinatol. (2015) 35:650–5. 10.1038/jp.2015.36 25927272 PMC4520760

[4] Desai RJ Hernandez-Diaz S Bateman BT Huybrechts KF. Increase in prescription opioid use during pregnancy among Medicaid-enrolled women. Obstet Gynecol. (2014) 123:997–1002. 10.1097/AOG.0000000000000208 24785852 PMC4020039

[5] Health USDo, Abuse HSS, Statistics MHSACfBH, Quality. National Survey on Drug Use and Health, 2012. Inter-university Consortium for Political and Social Research [distributor]. (2015). 10.3886/ICPSR34933.v3

[6] Hedegaard H Miniño AM Warner M. Drug Overdose Deaths in the United States, 1999–2019 NCHS Data Brief, (ed.) N.C.F.H. Hyatsville, MD: Statistics. (2020). 33395384

[7] Bio LL Siu A Poon CY. Update on the pharmacologic management of neonatal abstinence syndrome. J Perinatol. (2011) 31:692–701. 10.1038/jp.2011.116 21869765

[8] Jansson LM Velez M. Neonatal abstinence syndrome. Curr Opin Pediatr. (2012) 24:252–8. 10.1097/MOP.0b013e32834fdc3a 22227786

[9] Patrick SW Kaplan HC Passarella M Davis MM Lorch SA. Variation in treatment of neonatal abstinence syndrome in US Children's Hospitals, 2004–2011. J Perinatol. (2014) 34:867–72. 10.1038/jp.2014.114 24921412

[10] Grossman M Berkwitt A. Neonatal abstinence syndrome. Semin Perinatol. (2019) 43:173–86. 10.1053/j.semperi.2019.01.007 30773241

[11] Boggess T Risher WC. Clinical and basic research investigations into the long-term effects of prenatal opioid exposure on brain development. J Neurosci Res. (2020) 10.1002/jnr.24642. [Epub ahead of print]. 32459039

[12] Kayemba-Kay's S Laclyde JP. Buprenorphine withdrawal syndrome in newborns: a report of 13 cases. Addiction. (2003) 98:1599–604. 10.1046/j.1360-0443.2003.00551.x 14616186

[13] Burke S Beckwith AM. Morphine versus methadone treatment for neonatal withdrawal and impact on early infant development. Global Pediatric Health. (2017) 4:2333794X17721128-12333794X17721128. 10.1177/2333794X17721128 28804749 PMC5533256

[14] Smith RV Havens JR Walsh SL. Gabapentin misuse, abuse and diversion: a systematic review. Addiction (Abingdon, England). (2016) 111:1160–74. 10.1111/add.13324 27265421 PMC5573873

[15] Vickers Smith R Boland EM Young AM Lofwall MR Quiroz A Staton M. A qualitative analysis of gabapentin misuse and diversion among people who use drugs in Appalachian Kentucky. Psychol Addict Behav. (2018) 32:115–21. 10.1037/adb0000337 29239621 PMC5805633

[16] Baird CRW Fox P Colvin LA. Gabapentinoid abuse in order to potentiate the effect of methadone: a survey among substance misusers. Eur Addict Res. (2014) 20:115–8. 10.1159/000355268 24192603

[17] Bastiaens L Galus J Mazur C. Abuse of gabapentin is associated with opioid addiction. Psychiatric Quarterly. (2016) 87:763–7. 10.1007/s11126-016-9421-7 26887855

[18] Loudin S Murray S Prunty L Davies T Evans J Werthammer J. An atypical withdrawal syndrome in neonates prenatally exposed to gabapentin and opioids. J Pediatr. (2017) 181:286–8. 10.1016/j.jpeds.2016.11.004 27889067

[19] Wachtel SR Hu XT Galloway MP White FJ. D1 dopamine receptor stimulation enables the postsynaptic, but not autoreceptor, effects of D2 dopamine agonists in nigrostriatal and mesoaccumbens dopamine systems. Synapse. (1989) 4:327–46. 10.1002/syn.890040409 2532422

[20] Carlezon WAJr Wise RA. Rewarding actions of phencyclidine and related drugs in nucleus accumbens shell and frontal cortex. J Neurosci. (1996) 16:3112–22. 10.1523/JNEUROSCI.16-09-03112.1996 8622141 PMC6579051

[21] Di Chiara G Bassareo V Fenu S De Luca MA Spina L Cadoni C. Dopamine and drug addiction: the nucleus accumbens shell connection. Neuropharmacology. (2004) 47:227–41. 10.1016/j.neuropharm.2004.06.032 15464140

[22] Johnson S North R. Opioids excite dopamine neurons by hyperpolarization of local interneurons. J Neurosci. (1992) 12:483–8. 10.1523/JNEUROSCI.12-02-00483.1992 1346804 PMC6575608

[23] Han X Jing M-Y Zhao T-Y Wu N Song R Li J. Role of dopamine projections from ventral tegmental area to nucleus accumbens and medial prefrontal cortex in reinforcement behaviors assessed using optogenetic manipulation. Metab Brain Dis. (2017) 32:1491–502. 10.1007/s11011-017-0023-3 28523568

[24] Walton ME Bannerman DM Alterescu K Rushworth MFS. Functional specialization within medial frontal cortex of the anterior cingulate for evaluating effort-related decisions. J Neurosci. (2003) 23:6475–9. 10.1523/jneurosci.23-16-06475.2003 12878688 PMC6740644

[25] Blanchard TC Strait CE Hayden BY. Ramping ensemble activity in dorsal anterior cingulate neurons during persistent commitment to a decision. J Neurophysiol. (2015) 114:2439–49. 10.1152/jn.00711.2015 26334016 PMC4620134

[26] Elston TW Bilkey DK. Anterior cingulate cortex modulation of the ventral tegmental area in an effort task. Cell Rep. (2017) 19:2220–30. 10.1016/j.celrep.2017.05.062 28614710

[27] Narita M Matsushima Y Niikura K Narita M Takagi S Nakahara K. Implication of dopaminergic projection from the ventral tegmental area to the anterior cingulate cortex in μ-opioid-induced place preference. Addict Biol. (2010) 15:434–47. 10.1111/j.1369-1600.2010.00249.x 20731628

[28] Carr DB O'donnell P Card JP Sesack SR. Dopamine terminals in the rat prefrontal cortex synapse on pyramidal cells that project to the nucleus accumbens. J Neurosci. (1999) 19:11049–60. 10.1523/jneurosci.19-24-11049.1999 10594085 PMC6784921

[29] Mcfarland K Lapish CC Kalivas PW. Prefrontal glutamate release into the core of the nucleus accumbens mediates cocaine-induced reinstatement of drug-seeking behavior. J Neurophysiol. (2003) 23:3531–7. 10.1523/JNEUROSCI.23-08-03531.2003 12716962 PMC6742291

[30] Rebec GV Sun W. Neuronal substrates of relapse to cocaine-seeking behavior: role of prefrontal cortex. J Exp Anal Behav. (2005) 84:653–66. 10.1901/jeab.2005.105-04 16596984 PMC1389785

[31] Risher WC Kim N Koh S Choi J-E Mitev P Spence EF. Thrombospondin receptor α2δ-1 promotes synaptogenesis and spinogenesis via postsynaptic Rac1. J Cell Biol. (2018) 217:3747–65. 10.1083/jcb.201802057 30054448 PMC6168259

[32] Franklin K Paxinos G. (2008). The Mouse Brain in Stereotaxic Coordinates. New York, NY: Elsevier Inc.

[33] Ippolito DM Eroglu C. Quantifying synapses: an immunocytochemistry-based assay to quantify synapse number. J Vis Exp. (2010) 2270. 10.3791/2270 21113117 PMC3159596

[34] Eroglu C Allen NJ Susman MW O'rourke NA Park CY Ozkan E. Gabapentin receptor alpha2delta-1 is a neuronal thrombospondin receptor responsible for excitatory CNS synaptogenesis. Cell. (2009) 139:380–92. 10.1016/j.cell.2009.09.025 19818485 PMC2791798

[35] Field MJ Cox PJ Stott E Melrose H Offord J Su TZ. Identification of the alpha2-delta-1 subunit of voltage-dependent calcium channels as a molecular target for pain mediating the analgesic actions of pregabalin. Proc Natl Acad Sci U S A. (2006) 103:17537–42. 10.1073/pnas.0409066103 17088553 PMC1859964

[36] Chen J Li L Chen SR Chen H Xie JD Sirrieh RE. The alpha2delta-1-NMDA Receptor Complex Is Critically Involved in Neuropathic Pain Development and Gabapentin Therapeutic Actions. Cell Rep. (2018) 22:2307–21. 10.1016/j.celrep.2018.02.021 29490268 PMC5873963

[37] Heinsbroek JA De Vries TJ Peters J. Glutamatergic systems and memory mechanisms underlying opioid addiction. Cold Spring Harb Perspect Med. (2021) 11. 10.1101/cshperspect.a039602 32341068 PMC7718856

[38] Jalabert M Bourdy R Courtin J Veinante P Manzoni OJ Barrot M. Neuronal circuits underlying acute morphine action on dopamine neurons. Proc Nat Acad Sci. (2011) 201105418. 10.1073/pnas.1105418108 21930931 PMC3182694

[39] Lalumiere RT Kalivas PW. Glutamate release in the nucleus accumbens core is necessary for heroin seeking. J Neurosci. (2008) 28:3170–7. 10.1523/JNEUROSCI.5129-07.2008 18354020 PMC6670700

[40] Vaughan CW Ingram SL Connor MA Christie MJ. How opioids inhibit GABA-mediated neurotransmission. Nature. (1997) 390:611–4. 10.1038/37610 9403690

[41] Gardner EL. Addiction and brain reward and antireward pathways. Adv Psychosom Med. (2011) 30:22–60. 10.1159/000324065 21508625 PMC4549070

[42] Nygaard E Slinning K Moe V Due-Tonnessen P Fjell A Walhovd KB. Neuroanatomical characteristics of youths with prenatal opioid and poly-drug exposure. Neurotoxicol Teratol. (2018) 68:13–26. 10.1016/j.ntt.2018.04.004 29679636

[43] Labella MH Eiden RD Tabachnick AR Sellers T Dozier M. Infant neurodevelopmental outcomes of prenatal opioid exposure and polysubstance use. Neurotoxicol Teratol. (2021) 86:107000. 10.1016/j.ntt.2021.107000 34116198 PMC8277730

[44] Martin LB Thompson AC Martin T Kristal MB. Analgesic efficacy of orally administered buprenorphine in rats. Comp Med. (2001) 51:43–8. 11926301

[45] Kilic FS Sirmagul B Yildirim E Oner S Erol K. Antinociceptive effects of gabapentin and its mechanism of action in experimental animal studies. Indian J Med Res. (2012) 135:630–5. 22771591 PMC3401692

[46] De Felipe J Marco P Fairén A Jones EG. Inhibitory synaptogenesis in mouse somatosensory cortex. Cerebral Cortex. (1997) 7:619–34. 10.1093/cercor/7.7.619 9373018

[47] Christopherson KS Ullian EM Stokes CCA Mullowney CE Hell JW Agah A. Thrombospondins are astrocyte-secreted proteins that promote CNS synaptogenesis. Cell. (2005) 120:421–33. 10.1016/j.cell.2004.12.020 15707899

[48] Farhy-Tselnicker I Allen NJ. Astrocytes, neurons, synapses: a tripartite view on cortical circuit development. Neural Dev. (2018) 13:7–7. 29712572 10.1186/s13064-018-0104-y PMC5928581

[49] Otis EM Brent R. Equivalent ages in mouse and human embryos. Anat Rec. (1954) 120:33–63. 10.1186/s13064-018-0104-y 13207763

[50] Hill MA. Embryology Mouse Development [Online]. Sydney: University of New South Wales. (2020). Available online at: https://embryology.med.unsw.edu.au/embryology/index.php/Mouse_Development (accessed Feb 12, 2020).

[51] Kongstorp M Bogen IL Stiris T Andersen JM. High accumulation of methadone compared with buprenorphine in fetal rat brain after maternal exposure. J Pharmacol Exp Ther. (2019) 371:130–7. 10.1124/jpet.119.259531 31358559

[52] Ohman I Vitols S Tomson T. Pharmacokinetics of gabapentin during delivery, in the neonatal period, and lactation: does a fetal accumulation occur during pregnancy? Epilepsia. (2005) 46:1621–4. 10.1111/j.1528-1167.2005.00251.x 16190933

[53] Kristensen JH Ilett KF Hackett LP Kohan R. Gabapentin and breastfeeding: a case report. J Hum Lact. (2006) 22:426–8. 10.1177/0890334406293421 17062788

[54] Lindemalm S Nydert P Svensson JO Stahle L Sarman I. Transfer of buprenorphine into breast milk and calculation of infant drug dose. J Hum Lact. (2009) 25:199–205. 10.1177/0890334408328295 19136395

[55] Fink K Meder W Dooley DJ Göthert M. Inhibition of neuronal Ca(2+) influx by gabapentin and subsequent reduction of neurotransmitter release from rat neocortical slices. Br J Pharmacol. (2000) 130:900–6. 10.1038/sj.bjp.0703380 10864898 PMC1572136

[56] Patel R Dickenson AH. Mechanisms of the gabapentinoids and α 2 δ-1 calcium channel subunit in neuropathic pain. Pharmacol Res Perspect. (2016) 4. 10.1002/prp2.205 27069626 PMC4804325

[57] Nicolas S Gee JPB Visaka UK James D Richard O Geoffrey T. The novel anticonvulsant drug, gabapentin (Neurontin), binds to the α2δ Subunit of a calcium channel. J Biol Chem. (1996) 5768–76. 10.1074/jbc.271.10.5768 8621444

[58] Cole RL Lechner SM Williams ME Prodanovich P Bleicher L Varney MA. Differential distribution of voltage-gated calcium channel alpha-2 delta (α2δ) subunit mRNA-containing cells in the rat central nervous system and the dorsal root ganglia. J Comp Neurol. (2005) 491:246–69. 10.1002/cne.20693 16134135

[59] Nieto-Rostro M Ramgoolam K Pratt WS Kulik A Dolphin AC. Ablation of α_2_δ-1 inhibits cell-surface trafficking of endogenous N-type calcium channels in the pain pathway in vivo. Proc Nat Acad Sci. (2018) 115:E12043–52. 10.1073/pnas.1811212115 30487217 PMC6305000

[60] Tanabe T Takeshima H Mikami A Flockerzi V Takahashi H Kangawa K. Primary structure of the receptor for calcium channel blockers from skeletal muscle. Nature. (1987) 328:313–8. 10.1038/328313a0 3037387

[61] Catterall W Seagar M Takahashi M. Molecular properties of dihydropyridine-sensitive calcium channels in skeletal muscle. J Biol Chem. (1988) 263:3535–8. 10.1016/S0021-9258(18)68955-0 2450086

[62] Klugbauer N Lacinová L Marais E Hobom M Hofmann F. Molecular Diversity of the Calcium Channel α_2_δ Subunit. J Neurosci. (1999) 19:684–91. 10.1523/jneurosci.19-02-00684.1999 9880589 PMC6782206

[63] Ikeda H Miyatake M Koshikawa N Ochiai K Yamada K Kiss A. Morphine modulation of thrombospondin levels in astrocytes and its implications for neurite outgrowth and synapse formation. J Biol Chem. (2010) 285:38415–27. 10.1074/jbc.M110.109827 20889977 PMC2992274

[64] Phamduong E Rathore MK Crews NR D'angelo AS Leinweber AL Kappera P. Acute and chronic mu opioids differentially regulate thrombospondins 1 and 2 isoforms in astrocytes. ACS Chemical Neuroscience. (2014) 5:106–14. 10.1021/cn400172n 24304333 PMC3930990

[65] Cresto N Pillet L-E Billuart P Rouach N. Do Astrocytes play a role in intellectual disabilities? Trends Neurosci. (2019) 42:518–27. 10.1016/j.tins.2019.05.011 31300246

[66] Elmariah SB Oh EJ Hughes EG Balice-Gordon RJ. Astrocytes regulate inhibitory synapse formation via Trk-mediated modulation of postsynaptic GABAA receptors. The J Neurosci. (2005) 25:3638–50. 10.1523/JNEUROSCI.3980-04.2005 15814795 PMC6725365

[67] Chung W-S Clarke LE Wang GX Stafford BK Sher A Chakraborty C. Astrocytes mediate synapse elimination through MEGF10 and MERTK pathways. Nature. (2013) 504:394–400. 10.1038/nature12776 24270812 PMC3969024

[68] Iversen LL Neal MJ. The uptake of [3H]GABA by slices of rat cerebral cortex. J Neurochem. (1968) 15:1141–9. 10.1111/j.1471-4159.1968.tb06831.x 5711127

[69] Minelli A Debiasi S Brecha NC Zuccarello LV Conti F. GAT-3, a high-affinity GABA plasma membrane transporter, is localized to astrocytic processes, and it is not confined to the vicinity of GABAergic synapses in the cerebral cortex. J Neurochem. (1996) 16:6255–64. 10.1523/JNEUROSCI.16-19-06255.1996 8815906 PMC6579190

[70] Voutsinos-Porche B Bonvento G Tanaka K Steiner P Welker E Chatton J-Y. Glial glutamate transporters mediate a functional metabolic crosstalk between neurons and astrocytes in the mouse developing cortex. Neuron. (2003) 37:275–86. 10.1016/S0896-6273(02)01170-4 12546822

[71] Parpura V Haydon PG. Physiological astrocytic calcium levels stimulate glutamate release to modulate adjacent neurons. Proc Nat Acad Sci. (2000) 97:8629–34. 10.1073/PNAS.97.15.8629 10900020 PMC26999

[72] Boddum K Jensen TP Magloire V Kristiansen U Rusakov DA Pavlov I. Astrocytic GABA transporter activity modulates excitatory neurotransmission. Nat Commun. (2016) 7:13572. 10.1038/ncomms13572 27886179 PMC5133667

[73] Nam M-H Han K-S Lee J Bae JY An H Park S. Expression of μ-Opioid Receptor in CA1 Hippocampal Astrocytes. Exp Neurobiol. (2018) 27:120–8. 10.5607/en.2018.27.2.120 29731678 PMC5934543

[74] Hauser KF Stiene-Martin A Mattson MP Elde RP Ryan SE Godleske CC. mu-Opioid receptor-induced Ca2+ mobilization and astroglial development: morphine inhibits DNA synthesis and stimulates cellular hypertrophy through a Ca(2+)-dependent mechanism. Brain Res. (1996) 720:191–203. 10.1016/0006-8993(96)00103-5 8782912 PMC4338004

[75] Beitner-Johnson D Guitart X Nestler EJ. Glial fibrillary acidic protein and the mesolimbic dopamine system: regulation by chronic morphine and Lewis-Fischer strain differences in the rat ventral tegmental area. J Neurochem. (1993) 61:1766–73. 10.1111/j.1471-4159.1993.tb09814.x 8228992

