# Supplementary material for: Alterations in Excitatory and Inhibitory Synaptic Development Within the Mesolimbic Dopamine Pathway in a Mouse Model of Prenatal Drug Exposure
Source: Front Pediatr. 2021 Dec 13;9:794544. doi: 10.3389/fped.2021.794544 (PMC8710665; doi:10.3389/fped.2021.794544)
Supplement: Supplementary file 1 [file Data_Sheet_1.PDF]

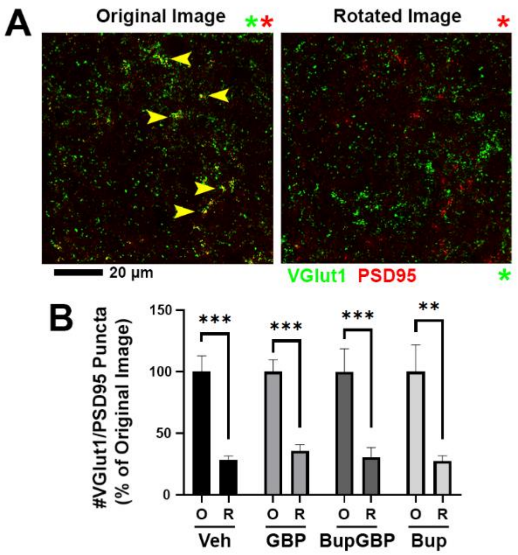

Supplementary Figure 1: Quantification of synaptic puncta depends on close spatial association between pre- and postsynaptic markers. **A)** Representative excitatory synaptic labeling (VGlut1, green; PSD95 red) in the ACC. The original image is shown on the left, with numerous sites of co-localized fluorescent labeling indicating sites of excitatory synapses (yellow, arrowheads). By rotating just the green (VGlut1) channel clockwise by 90 degrees (the colored asterisks indicate where the corresponding corners appear in each image), the rotated image on the right shows almost no co-localization despite the same density of fluorescent labeling. **B)** Fold-change comparison of co-localized VGlut1/PSD95 puncta between original (O) and rotated (R) images for 10 randomly selected images per treatment condition from the WT dataset analyzed in this study. In each case, the rotated image group had less than 30% of the number of quantified puncta compared to the original (non-rotated) image. ** $p < .01$; *** $p < .001$; multiple paired t-tests (vehicle: $t(9) = 5.743$, $p = .0003$; GBP: $t(9) = 5.828$, $p = .0003$; Bup+GBP: $t(9) = 5.316$, $p = .0005$; Bup: $t(9) = 3.918$, $p = .0035$).

Type	Group 1	Group 2	Significance	Adjusted *p*
Pups	Vehicle/WT M	Vehicle/WT F	ns	0.5326
	Vehicle/WT M	Vehicle/Het M	ns	>0.9999
	Vehicle/WT M	Vehicle/Het F	ns	0.8959
	Vehicle/WT M	Gabapentin/WT M	ns	0.7536
	Vehicle/WT M	Gabapentin/WT F	ns	>0.9999
	Vehicle/WT M	Gabapentin/Het M	ns	>0.9999
	Vehicle/WT M	Gabapentin/Het F	ns	0.9977
	Vehicle/WT M	Buprenorphine/WT M	****	<0.0001
	Vehicle/WT M	Buprenorphine/WT F	***	0.0006
	Vehicle/WT M	Buprenorphine/Het M	*	0.0112
	Vehicle/WT M	Buprenorphine/Het F	ns	0.1152
	Vehicle/WT M	Bup + GBP/WT M	**	0.0073
	Vehicle/WT M	Bup + GBP/WT F	**	0.0018
	Vehicle/WT M	Bup + GBP/Het M	****	<0.0001
	Vehicle/WT M	Bup + GBP/Het F	**	0.0015
	Vehicle/WT F	Vehicle/Het M	ns	0.1394
	Vehicle/WT F	Vehicle/Het F	ns	>0.9999
	Vehicle/WT F	Gabapentin/WT M	ns	>0.9999
	Vehicle/WT F	Gabapentin/WT F	ns	0.4393
	Vehicle/WT F	Gabapentin/Het M	ns	0.1434
	Vehicle/WT F	Gabapentin/Het F	ns	0.9905
	Vehicle/WT F	Buprenorphine/WT M	**	0.0035
	Vehicle/WT F	Buprenorphine/WT F	ns	0.1772
	Vehicle/WT F	Buprenorphine/Het M	ns	0.8616
	Vehicle/WT F	Buprenorphine/Het F	ns	0.9991
	Vehicle/WT F	Bup + GBP/WT M	****	<0.0001
	Vehicle/WT F	Bup + GBP/WT F	****	<0.0001
	Vehicle/WT F	Bup + GBP/Het M	****	<0.0001
	Vehicle/WT F	Bup + GBP/Het F	****	<0.0001
	Vehicle/Het M	Vehicle/Het F	ns	0.4262
	Vehicle/Het M	Gabapentin/WT M	ns	0.4018
	Vehicle/Het M	Gabapentin/WT F	ns	>0.9999
	Vehicle/Het M	Gabapentin/Het M	ns	>0.9999
	Vehicle/Het M	Gabapentin/Het F	ns	0.9122
	Vehicle/Het M	Buprenorphine/WT M	****	<0.0001
	Vehicle/Het M	Buprenorphine/WT F	****	<0.0001
	Vehicle/Het M	Buprenorphine/Het M	***	0.0005
	Vehicle/Het M	Buprenorphine/Het F	*	0.0158
	Vehicle/Het M	Bup + GBP/WT M	**	0.0044

	Vehicle/Het M	Bup + GBP/WT F	**	0.001
	Vehicle/Het M	Bup + GBP/Het M	****	<0.0001
	Vehicle/Het M	Bup + GBP/Het F	***	0.0009
	Vehicle/Het F	Gabapentin/WT M	ns	>0.9999
	Vehicle/Het F	Gabapentin/WT F	ns	0.8463
	Vehicle/Het F	Gabapentin/Het M	ns	0.405
	Vehicle/Het F	Gabapentin/Het F	ns	>0.9999
	Vehicle/Het F	Buprenorphine/WT M	****	<0.0001
	Vehicle/Het F	Buprenorphine/WT F	*	0.0127
	Vehicle/Het F	Buprenorphine/Het M	ns	0.2241
	Vehicle/Het F	Buprenorphine/Het F	ns	0.8218
	Vehicle/Het F	Bup + GBP/WT M	****	<0.0001
	Vehicle/Het F	Bup + GBP/WT F	****	<0.0001
	Vehicle/Het F	Bup + GBP/Het M	****	<0.0001
	Vehicle/Het F	Bup + GBP/Het F	****	<0.0001
	Gabapentin/WT M	Gabapentin/WT F	ns	0.7105
	Gabapentin/WT M	Gabapentin/Het M	ns	0.3451
	Gabapentin/WT M	Gabapentin/Het F	ns	0.9978
	Gabapentin/WT M	Buprenorphine/WT M	*	0.0346
	Gabapentin/WT M	Buprenorphine/WT F	ns	0.4755
	Gabapentin/WT M	Buprenorphine/Het M	ns	0.9784
	Gabapentin/WT M	Buprenorphine/Het F	ns	>0.9999
	Gabapentin/WT M	Bup + GBP/WT M	****	<0.0001
	Gabapentin/WT M	Bup + GBP/WT F	****	<0.0001
	Gabapentin/WT M	Bup + GBP/Het M	****	<0.0001
	Gabapentin/WT M	Bup + GBP/Het F	****	<0.0001
	Gabapentin/WT F	Gabapentin/Het M	ns	>0.9999
	Gabapentin/WT F	Gabapentin/Het F	ns	0.9967
	Gabapentin/WT F	Buprenorphine/WT M	****	<0.0001
	Gabapentin/WT F	Buprenorphine/WT F	***	0.0002
	Gabapentin/WT F	Buprenorphine/Het M	**	0.005
	Gabapentin/WT F	Buprenorphine/Het F	ns	0.0764
	Gabapentin/WT F	Bup + GBP/WT M	**	0.0022
	Gabapentin/WT F	Bup + GBP/WT F	***	0.0005
	Gabapentin/WT F	Bup + GBP/Het M	****	<0.0001
	Gabapentin/WT F	Bup + GBP/Het F	***	0.0005
	Gabapentin/Het M	Gabapentin/Het F	ns	0.8452
	Gabapentin/Het M	Buprenorphine/WT M	****	<0.0001
	Gabapentin/Het M	Buprenorphine/WT F	****	<0.0001
	Gabapentin/Het M	Buprenorphine/Het M	**	0.0012
	Gabapentin/Het M	Buprenorphine/Het F	*	0.0196

	Gabapentin/Het M	Bup + GBP/WT M	ns	0.0563
	Gabapentin/Het M	Bup + GBP/WT F	*	0.0142
	Gabapentin/Het M	Bup + GBP/Het M	***	0.0002
	Gabapentin/Het M	Bup + GBP/Het F	*	0.0124
	Gabapentin/Het F	Buprenorphine/WT M	****	<0.0001
	Gabapentin/Het F	Buprenorphine/WT F	**	0.0073
	Gabapentin/Het F	Buprenorphine/Het M	ns	0.1242
	Gabapentin/Het F	Buprenorphine/Het F	ns	0.601
	Gabapentin/Het F	Bup + GBP/WT M	****	<0.0001
	Gabapentin/Het F	Bup + GBP/WT F	****	<0.0001
	Gabapentin/Het F	Bup + GBP/Het M	****	<0.0001
	Gabapentin/Het F	Bup + GBP/Het F	****	<0.0001
	Buprenorphine/WT M	Buprenorphine/WT F	ns	0.9864
	Buprenorphine/WT M	Buprenorphine/Het M	ns	0.2873
	Buprenorphine/WT M	Buprenorphine/Het F	ns	0.1152
	Buprenorphine/WT M	Bup + GBP/WT M	****	<0.0001
	Buprenorphine/WT M	Bup + GBP/WT F	****	<0.0001
	Buprenorphine/WT M	Bup + GBP/Het M	****	<0.0001
	Buprenorphine/WT M	Bup + GBP/Het F	****	<0.0001
	Buprenorphine/WT F	Buprenorphine/Het M	ns	0.9921
	Buprenorphine/WT F	Buprenorphine/Het F	ns	0.8703
	Buprenorphine/WT F	Bup + GBP/WT M	****	<0.0001
	Buprenorphine/WT F	Bup + GBP/WT F	****	<0.0001
	Buprenorphine/WT F	Bup + GBP/Het M	****	<0.0001
	Buprenorphine/WT F	Bup + GBP/Het F	****	<0.0001
	Buprenorphine/Het M	Buprenorphine/Het F	ns	>0.9999
	Buprenorphine/Het M	Bup + GBP/WT M	****	<0.0001
	Buprenorphine/Het M	Bup + GBP/WT F	****	<0.0001
	Buprenorphine/Het M	Bup + GBP/Het M	****	<0.0001
	Buprenorphine/Het M	Bup + GBP/Het F	****	<0.0001
	Buprenorphine/Het F	Bup + GBP/WT M	****	<0.0001
	Buprenorphine/Het F	Bup + GBP/WT F	****	<0.0001
	Buprenorphine/Het F	Bup + GBP/Het M	****	<0.0001
	Buprenorphine/Het F	Bup + GBP/Het F	****	<0.0001
	Bup + GBP/WT M	Bup + GBP/WT F	ns	>0.9999
	Bup + GBP/WT M	Bup + GBP/Het M	ns	0.7611
	Bup + GBP/WT M	Bup + GBP/Het F	ns	>0.9999
	Bup + GBP/WT F	Bup + GBP/Het M	ns	0.9985
	Bup + GBP/WT F	Bup + GBP/Het F	ns	>0.9999
	Bup + GBP/Het M	Bup + GBP/Het F	ns	0.9991
**Dams**	Vehicle	Gabapentin	***	0.0006

	Vehicle	Buprenorphine	*	0.0122
	Vehicle	Bup + GBP	****	<0.0001
	Gabapentin	Buprenorphine	ns	0.5055
	Gabapentin	Bup + GBP	****	<0.0001
	Buprenorphine	Bup + GBP	****	<0.0001

Supplementary Table 1: Complete results of Tukey's multiple comparisons analysis following ANOVA testing of body mass differences amongst treatment groups for dams and pups used in the study. * $p < .05$; ** $p < .01$; *** $p < .001$; **** $p < .0001$; "ns" = not significant

Synapse	Region	Genotype	Treatment	K2	Significance	*p*-value	Passed Normality Test?
Excitatory	ACC	WT	Veh	42.72	****	<0.0001	no
			GBP	49.31	****	<0.0001	no
			BupGBP	14.65	***	0.0007	no
			Bup	12.26	**	0.0022	no
		Het	Veh	0.20	ns	0.9048	yes
			GBP	33.22	****	<0.0001	no
			BupGBP	20.63	****	<0.0001	no
			Bup	14.20	***	0.0008	no
	NAC	WT	Veh	26.84	****	<0.0001	no
			GBP	47.50	****	<0.0001	no
			BupGBP	19.34	****	<0.0001	no
			Bup	12.97	**	0.0015	no
		Het	Veh	39.16	****	<0.0001	no
			GBP	30.03	****	<0.0001	no
			BupGBP	24.75	****	<0.0001	no
			Bup	13.54	**	0.0011	no
	PFC	WT	Veh	16.04	***	0.0003	no
			GBP	52.13	****	<0.0001	no
			BupGBP	14.29	***	0.0008	no
			Bup	9.07	*	0.0107	no
		Het	Veh	13.29	**	0.0013	no
			GBP	26.36	****	<0.0001	no
			BupGBP	28.78	****	<0.0001	no
			Bup	15.43	***	0.0004	no
Inhibitory	ACC	WT	Veh	10.43	**	0.0054	no
			GBP	4.23	ns	0.1208	yes
			BupGBP	41.02	****	<0.0001	no
			Bup	6.48	*	0.0392	no
		Het	Veh	39.31	****	<0.0001	no
			GBP	6.70	*	0.0351	no
			BupGBP	19.76	****	<0.0001	no
			Bup	26.00	****	<0.0001	no
	NAC	WT	Veh	8.02	*	0.0181	no
			GBP	7.23	*	0.0269	no
			BupGBP	25.97	****	<0.0001	no
			Bup	1.87	ns	0.3936	yes
		Het	Veh	57.06	****	<0.0001	no
			GBP	2.95	ns	0.2286	yes
			BupGBP	28.85	****	<0.0001	no

			Bup	15.12	***	0.0005	no
	PFC	WT	Veh	5.56	ns	0.062	yes
			GBP	5.38	ns	0.0678	yes
			BupGBP	26.44	****	<0.0001	no
			Bup	10.92	**	0.0043	no
		Het	Veh	56.10	****	<0.0001	no
			GBP	1.03	ns	0.5975	yes
			BupGBP	20.72	****	<0.0001	no
			Bup	22.92	****	<0.0001	no

Supplementary Table 2: Complete results of D'Agostino and Pearson omnibus normality testing on excitatory and inhibitory synaptic puncta datasets analyzed in this study. * $p < .05$; ** $p < .01$; *** $p < .001$; **** $p < .0001$; "ns" = not significant

Excitatory Synapses (VGlut1/PSD95 Co-localized Puncta)				
Region	Group 1	Group 2	Significance	Adjusted *p*
ACC	WT Vehicle	WT Gabapentin	ns	0.2403
	WT Vehicle	WT Bup+GBP	****	<0.0001
	WT Vehicle	WT Buprenorphine	***	0.0004
	WT Gabapentin	WT Bup+GBP	ns	>0.9999
	WT Gabapentin	WT Buprenorphine	ns	>0.9999
	WT Bup+GBP	WT Buprenorphine	ns	>0.9999
	WT Vehicle	Het Vehicle	ns	0.1278
	WT Vehicle	Het Gabapentin	****	<0.0001
	WT Vehicle	Het Bup+GBP	ns	>0.9999
	WT Vehicle	Het Buprenorphine	****	<0.0001
	WT Gabapentin	Het Vehicle	ns	>0.9999
	WT Gabapentin	Het Gabapentin	****	<0.0001
	WT Gabapentin	Het Bup+GBP	ns	>0.9999
	WT Gabapentin	Het Buprenorphine	**	0.0017
	WT Bup+GBP	Het Vehicle	ns	>0.9999
	WT Bup+GBP	Het Gabapentin	**	0.0091
	WT Bup+GBP	Het Bup+GBP	ns	0.182
	WT Bup+GBP	Het Buprenorphine	ns	0.8729
	WT Buprenorphine	Het Vehicle	ns	>0.9999
	WT Buprenorphine	Het Gabapentin	**	0.009
	WT Buprenorphine	Het Bup+GBP	ns	0.3724
	WT Buprenorphine	Het Buprenorphine	ns	0.7524
	Het Vehicle	Het Gabapentin	****	<0.0001
	Het Vehicle	Het Bup+GBP	ns	>0.9999
	Het Vehicle	Het Buprenorphine	***	0.0007
	Het Gabapentin	Het Bup+GBP	****	<0.0001
	Het Gabapentin	Het Buprenorphine	ns	>0.9999
	Het Bup+GBP	Het Buprenorphine	****	<0.0001
NAC	WT Vehicle	WT Gabapentin	ns	>0.9999
	WT Vehicle	WT Bup+GBP	**	0.0013
	WT Vehicle	WT Buprenorphine	****	<0.0001
	WT Gabapentin	WT Bup+GBP	***	0.0001
	WT Gabapentin	WT Buprenorphine	****	<0.0001
	WT Bup+GBP	WT Buprenorphine	ns	>0.9999
	WT Vehicle	Het Vehicle	ns	>0.9999
	WT Vehicle	Het Gabapentin	****	<0.0001
	WT Vehicle	Het Bup+GBP	ns	0.4958
	WT Vehicle	Het Buprenorphine	****	<0.0001

	WT Gabapentin	Het Vehicle	ns	0.4165
	WT Gabapentin	Het Gabapentin	****	<0.0001
	WT Gabapentin	Het Bup+GBP	ns	0.0937
	WT Gabapentin	Het Buprenorphine	****	<0.0001
	WT Bup+GBP	Het Vehicle	ns	0.4885
	WT Bup+GBP	Het Gabapentin	**	0.0017
	WT Bup+GBP	Het Bup+GBP	ns	>0.9999
	WT Bup+GBP	Het Buprenorphine	ns	>0.9999
	WT Buprenorphine	Het Vehicle	*	0.0432
	WT Buprenorphine	Het Gabapentin	ns	0.0608
	WT Buprenorphine	Het Bup+GBP	ns	0.3555
	WT Buprenorphine	Het Buprenorphine	ns	>0.9999
	Het Vehicle	Het Gabapentin	****	<0.0001
	Het Vehicle	Het Bup+GBP	ns	>0.9999
	Het Vehicle	Het Buprenorphine	**	0.0046
	Het Gabapentin	Het Bup+GBP	****	<0.0001
	Het Gabapentin	Het Buprenorphine	ns	0.1743
	Het Bup+GBP	Het Buprenorphine	ns	0.066
**PFC**	WT Vehicle	WT Gabapentin	ns	>0.9999
	WT Vehicle	WT Bup+GBP	ns	>0.9999
	WT Vehicle	WT Buprenorphine	ns	0.3807
	WT Gabapentin	WT Bup+GBP	ns	>0.9999
	WT Gabapentin	WT Buprenorphine	ns	0.4236
	WT Bup+GBP	WT Buprenorphine	ns	0.0916
	WT Vehicle	Het Vehicle	ns	0.5396
	WT Vehicle	Het Gabapentin	****	<0.0001
	WT Vehicle	Het Bup+GBP	ns	0.0923
	WT Vehicle	Het Buprenorphine	****	<0.0001
	WT Gabapentin	Het Vehicle	ns	0.4802
	WT Gabapentin	Het Gabapentin	****	<0.0001
	WT Gabapentin	Het Bup+GBP	ns	0.0812
	WT Gabapentin	Het Buprenorphine	****	<0.0001
	WT Bup+GBP	Het Vehicle	ns	>0.9999
	WT Bup+GBP	Het Gabapentin	****	<0.0001
	WT Bup+GBP	Het Bup+GBP	ns	0.2156
	WT Bup+GBP	Het Buprenorphine	****	<0.0001
	WT Buprenorphine	Het Vehicle	****	<0.0001
	WT Buprenorphine	Het Gabapentin	ns	0.3221
	WT Buprenorphine	Het Bup+GBP	****	<0.0001
	WT Buprenorphine	Het Buprenorphine	*	0.0275
	Het Vehicle	Het Gabapentin	****	<0.0001

	Het Vehicle	Het Bup+GBP	ns	>0.9999
	Het Vehicle	Het Buprenorphine	****	<0.0001
	Het Gabapentin	Het Bup+GBP	****	<0.0001
	Het Gabapentin	Het Buprenorphine	ns	>0.9999
	Het Bup+GBP	Het Buprenorphine	****	<0.0001

Supplementary Table 3: Complete results of Dunn's multiple comparisons analysis following Kruskal-Wallis testing of co-localized excitatory synaptic puncta counts, organized by brain region (ACC/NAC/PFC). * $p < .05$; ** $p < .01$; *** $p < .001$; **** $p < .0001$; "ns" = not significant

Inhibitory Synapses (VGAT/Gephyrin Co-localized Puncta)				
Region	Group 1	Group 2	Significance	Adjusted *p*
ACC	WT Vehicle	WT Gabapentin	ns	>0.9999
	WT Vehicle	WT Bup+GBP	****	<0.0001
	WT Vehicle	WT Buprenorphine	ns	0.3148
	WT Gabapentin	WT Bup+GBP	****	<0.0001
	WT Gabapentin	WT Buprenorphine	ns	>0.9999
	WT Bup+GBP	WT Buprenorphine	****	<0.0001
	WT Vehicle	Het Vehicle	****	<0.0001
	WT Vehicle	Het Gabapentin	****	<0.0001
	WT Vehicle	Het Bup+GBP	*	0.0281
	WT Vehicle	Het Buprenorphine	*	0.0479
	WT Gabapentin	Het Vehicle	****	<0.0001
	WT Gabapentin	Het Gabapentin	****	<0.0001
	WT Gabapentin	Het Bup+GBP	**	0.0055
	WT Gabapentin	Het Buprenorphine	*	0.0101
	WT Bup+GBP	Het Vehicle	ns	>0.9999
	WT Bup+GBP	Het Gabapentin	ns	>0.9999
	WT Bup+GBP	Het Bup+GBP	****	<0.0001
	WT Bup+GBP	Het Buprenorphine	**	0.0063
	WT Buprenorphine	Het Vehicle	****	<0.0001
	WT Buprenorphine	Het Gabapentin	****	<0.0001
	WT Buprenorphine	Het Bup+GBP	****	<0.0001
	WT Buprenorphine	Het Buprenorphine	****	<0.0001
	Het Vehicle	Het Gabapentin	ns	>0.9999
	Het Vehicle	Het Bup+GBP	****	<0.0001
	Het Vehicle	Het Buprenorphine	*	0.0168
	Het Gabapentin	Het Bup+GBP	ns	0.4519
	Het Gabapentin	Het Buprenorphine	ns	>0.9999
	Het Bup+GBP	Het Buprenorphine	ns	>0.9999
NAC	WT Vehicle	WT Gabapentin	ns	>0.9999
	WT Vehicle	WT Bup+GBP	****	<0.0001
	WT Vehicle	WT Buprenorphine	ns	0.9649
	WT Gabapentin	WT Bup+GBP	****	<0.0001
	WT Gabapentin	WT Buprenorphine	ns	0.1903
	WT Bup+GBP	WT Buprenorphine	****	<0.0001
	WT Vehicle	Het Vehicle	****	<0.0001
	WT Vehicle	Het Gabapentin	****	<0.0001
	WT Vehicle	Het Bup+GBP	****	<0.0001
	WT Vehicle	Het Buprenorphine	****	<0.0001

	WT Gabapentin	Het Vehicle	****	<0.0001
	WT Gabapentin	Het Gabapentin	**	0.0044
	WT Gabapentin	Het Bup+GBP	****	<0.0001
	WT Gabapentin	Het Buprenorphine	****	<0.0001
	WT Bup+GBP	Het Vehicle	ns	>0.9999
	WT Bup+GBP	Het Gabapentin	ns	0.5578
	WT Bup+GBP	Het Bup+GBP	ns	>0.9999
	WT Bup+GBP	Het Buprenorphine	ns	0.1089
	WT Buprenorphine	Het Vehicle	****	<0.0001
	WT Buprenorphine	Het Gabapentin	****	<0.0001
	WT Buprenorphine	Het Bup+GBP	****	<0.0001
	WT Buprenorphine	Het Buprenorphine	****	<0.0001
	Het Vehicle	Het Gabapentin	ns	>0.9999
	Het Vehicle	Het Bup+GBP	ns	>0.9999
	Het Vehicle	Het Buprenorphine	**	0.0011
	Het Gabapentin	Het Bup+GBP	ns	>0.9999
	Het Gabapentin	Het Buprenorphine	****	<0.0001
	Het Bup+GBP	Het Buprenorphine	***	0.0003
**PFC**	WT Vehicle	WT Gabapentin	ns	>0.9999
	WT Vehicle	WT Bup+GBP	ns	0.0793
	WT Vehicle	WT Buprenorphine	**	0.0011
	WT Gabapentin	WT Bup+GBP	***	0.0004
	WT Gabapentin	WT Buprenorphine	ns	0.3935
	WT Bup+GBP	WT Buprenorphine	****	<0.0001
	WT Vehicle	Het Vehicle	*	0.0423
	WT Vehicle	Het Gabapentin	ns	>0.9999
	WT Vehicle	Het Bup+GBP	ns	>0.9999
	WT Vehicle	Het Buprenorphine	****	<0.0001
	WT Gabapentin	Het Vehicle	***	0.0001
	WT Gabapentin	Het Gabapentin	ns	>0.9999
	WT Gabapentin	Het Bup+GBP	ns	0.1562
	WT Gabapentin	Het Buprenorphine	****	<0.0001
	WT Bup+GBP	Het Vehicle	ns	>0.9999
	WT Bup+GBP	Het Gabapentin	ns	0.0549
	WT Bup+GBP	Het Bup+GBP	ns	>0.9999
	WT Bup+GBP	Het Buprenorphine	**	0.0057
	WT Buprenorphine	Het Vehicle	****	<0.0001
	WT Buprenorphine	Het Gabapentin	*	0.0123
	WT Buprenorphine	Het Bup+GBP	****	<0.0001
	WT Buprenorphine	Het Buprenorphine	****	<0.0001
	Het Vehicle	Het Gabapentin	*	0.0338

	Het Vehicle	Het Bup+GBP	ns	>0.9999
	Het Vehicle	Het Buprenorphine	***	0.0005
	Het Gabapentin	Het Bup+GBP	ns	>0.9999
	Het Gabapentin	Het Buprenorphine	****	<0.0001
	Het Bup+GBP	Het Buprenorphine	****	<0.0001

Supplementary Table 4: Complete results of Dunn’s multiple comparisons analysis following Kruskal-Wallis testing of co-localized inhibitory synaptic puncta counts, organized by brain region (ACC/NAC/PFC). * $p < .05$; ** $p < .01$; *** $p < .001$; **** $p < .0001$; “ns” = not significant
